# Anti-PD-1 therapy triggers Tfh cell–dependent IL-4 release to boost CD8 T cell responses in tumor-draining lymph nodes

**DOI:** 10.1084/jem.20232104

**Published:** 2024-02-28

**Authors:** Mathilde Ruggiu, Marion V. Guérin, Béatrice Corre, Margot Bardou, Ruby Alonso, Erica Russo, Zacarias Garcia, Lea Feldmann, Fabrice Lemaître, Mathilde Dusseaux, Capucine L. Grandjean, Philippe Bousso

**Affiliations:** 1 Institut Pasteur, Université de Paris Cité, INSERM U1223, Paris, France; 2 Human Disease Models Core Facility, Institut Pasteur, Paris, France; 3 Vaccine Research Institute, Creteil, France

## Abstract

Anti-PD-1 therapy targets intratumoral CD8^+^ T cells to promote clinical responses in cancer patients. Recent evidence suggests an additional activity in the periphery, but the underlying mechanism is unclear. Here, we show that anti-PD-1 mAb enhances CD8^+^ T cell responses in tumor-draining lymph nodes by stimulating cytokine production in follicular helper T cells (Tfh). In two different models, anti-PD-1 mAb increased the activation and proliferation of tumor-specific T cells in lymph nodes. Surprisingly, anti-PD-1 mAb did not primarily target CD8^+^ T cells but instead stimulated IL-4 production by Tfh cells, the major population bound by anti-PD-1 mAb. Blocking IL-4 or inhibiting the Tfh master transcription factor BCL6 abrogated anti-PD-1 mAb activity in lymph nodes while injection of IL-4 complexes was sufficient to recapitulate anti-PD-1 mAb activity. A similar mechanism was observed in a vaccine model. Finally, nivolumab also boosted human Tfh cells in humanized mice. We propose that Tfh cells and IL-4 play a key role in the peripheral activity of anti-PD-1 mAb.

## Teaser

The peripheral activity of anti-PD-1 mAb is not fully understood. Here, Ruggiu, Guérin et al. demonstrate that anti-PD-1 mAb promotes CD8^+^ T cell responses in tumor-draining lymph node by targeting Tfh cells and stimulating IL-4 production.

## Graphical Abstract

**Figure:**
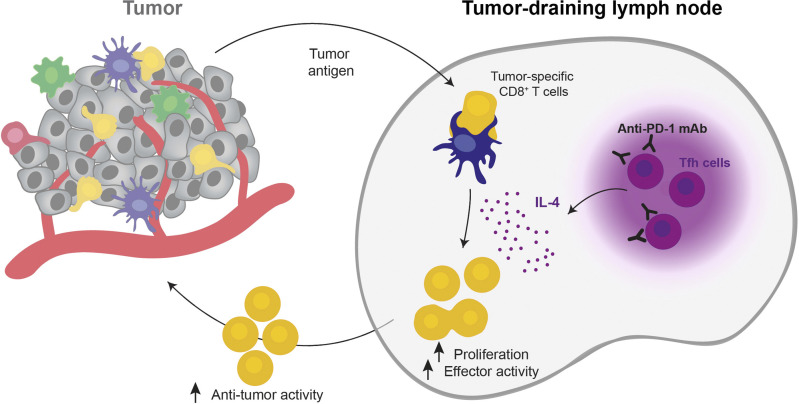

## Introduction

Blockade of the PD-1/PD-L1 axis has demonstrated a strong activity during treatment of several cancers, including melanoma (Robert et al., 2015; Weber et al., 2015), non-small-cell lung cancer (Herbst et al., 2016; Reck et al., 2016), and hematologic malignancies such as Hodgkin lymphoma (Armand et al., 2016). However, some patients do not respond or relapse after anti-PD-1 monoclonal antibody (mAb) (Ribas and Wolchok, 2018), highlighting the need for a better understanding of its mode of action in vivo.

It is well-established that anti-PD-1 mAb acts in the tumor microenvironment (TME) (Ribas and Wolchok, 2018). Upon binding to tumor-infiltrating CD8^+^ T cells (Arlauckas et al., 2017), it interferes with PD-1 binding to its ligands PD-L1 and PD-L2, expressed by tumor cells or antigen-presenting cells, including dendritic cells (DCs) (Garris et al., 2018; Oh et al., 2020; Peng et al., 2020; Waldman et al., 2020). Consequently, anti-PD-1 mAb promotes the expansion and differentiation of “stem-like” TCF-1^+^ PD-1^+^ CD8^+^ T cells that are essential for antitumor activity (Brummelman et al., 2018; Im et al., 2016; Kurtulus et al., 2019; Sade-Feldman et al., 2018; Siddiqui et al., 2019; Utzschneider et al., 2016).

Interestingly, recent evidence suggests that PD-1 blockade may not only act at the tumor site but display additional activity in the periphery (Spitzer et al., 2017; van der Leun et al., 2020; Wu et al., 2020). In line with this idea, anti-PD-1 mAb has been shown to promote the emergence of new clonotypes of tumor-specific CD8^+^ T cells in preclinical models and patient tumor biopsies (Liu et al., 2022; Nagasaki et al., 2022; Wu et al., 2020; Yost et al., 2019). In addition, blocking lymph node egress with FTY720 or surgically removing of the tumor-draining lymph node reduced the overall antitumor activity elicited by PD-1/PD-L1 blockade in mice (Dammeijer et al., 2020; Fransen et al., 2018; Nagasaki et al., 2022). Finally, a profound remodeling of the cellular composition of the tumor-draining lymph node has been observed in mice treated with anti-PD-1 mAb (Ho et al., 2020).

While these recent studies highlighted a contribution of lymph node cells during PD-1 blockade, the underlying mechanism remains largely unknown. For example, it is unclear whether anti-PD-1 mAb diffuses efficiently in lymph nodes, binds to tumor-specific CD8^+^ T cells, or relies on additional cellular and molecular players to mediate its antitumor activity.

Here, we show that anti-PD-1 mAb promotes the magnitude and quality of antigen-specific CD8^+^ T cell responses in the draining lymph node. Remarkably, we find that anti-PD-1 mAb initially targets follicular helper T cells (Tfh), thus eliciting IL-4 production to enhance CD8^+^ T cell activation and expansion. We provide evidence that CD8^+^ T cells expanded through the Tfh/IL-4 axis contribute to anti-tumor activity. A similar IL-4–dependent promotion of CD8^+^ T cell responses by anti-PD-1 mAb was also characterized in a vaccine model, suggesting a general mechanism for the peripheral activity of PD-1 checkpoint blockade.

## Results

### Anti-PD-1 mAb promotes tumor-specific CD8^+^ T cell expansion in draining lymph nodes

**Figure 1.**
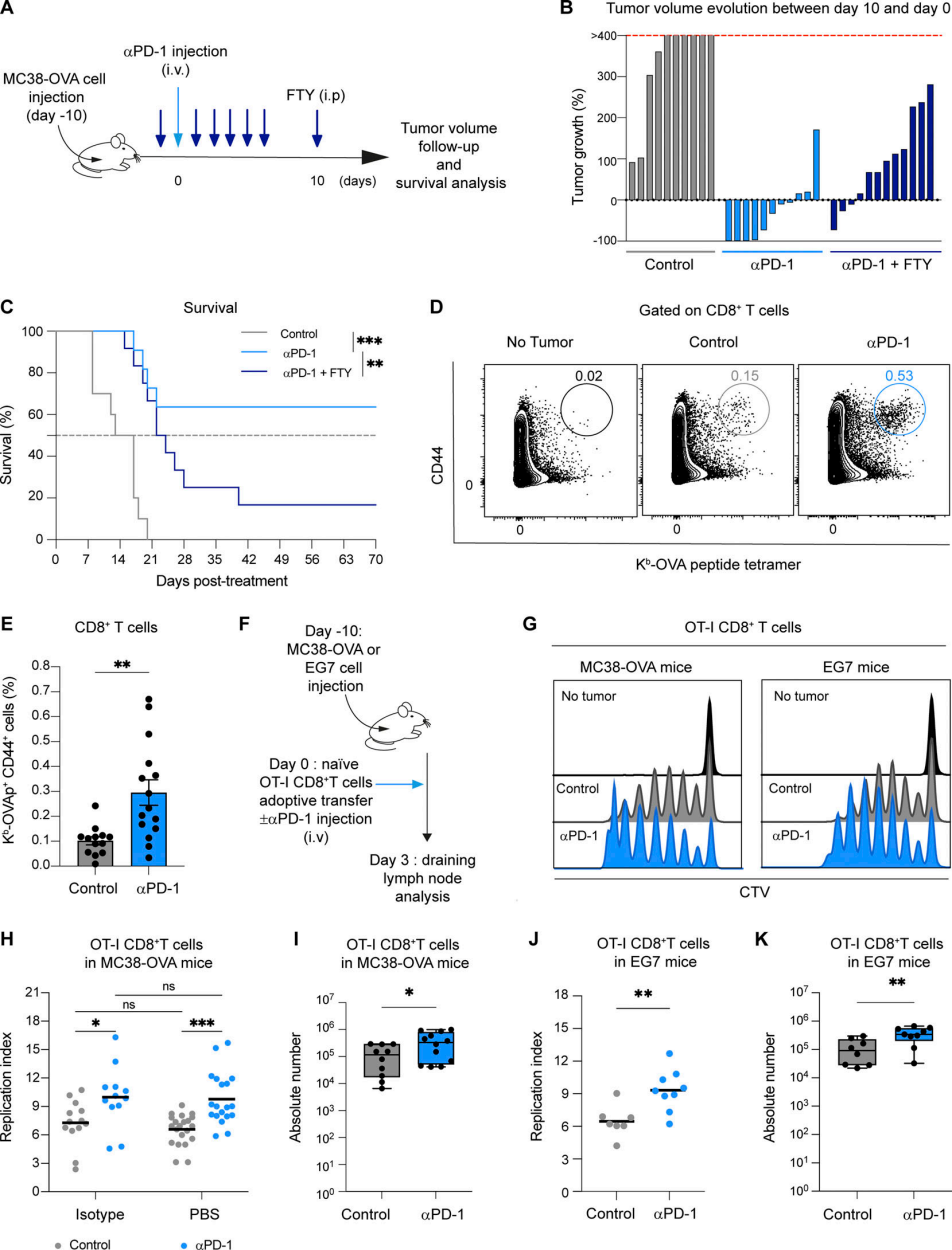
**Anti-PD-1 mAb promotes tumor-specific CD8**^**+**^ **T cell proliferation in draining lymph nodes. (A–C)** C57BL/6 mice were injected s.c. with MC38-OVA tumor cells (0.5 × 10^6^ cells). After 10 days, mice were treated or not with anti-PD-1 (250 µg, i.v.) either alone or anti-PD-1 in combination with FTY720 (20 µg for each injection). **(A)** Experimental setup. **(B)** Evolution of tumor volume between day 0 and day 10. **(C)** Mice survival. Statistical analysis was performed using a log-rank test. Compiled from two independent experiments with 10–12 mice per group. **(D and E)** Representative FACS contour plots and quantification of H2-K^b^-OVAp tetramers^+^ among CD8^+^ T cells 5 days after treatment. Tumor-free mice were included as a control. Compiled from five independent experiments with 13–15 mice per group. **(F–I)** C57BL/6 mice were injected s.c. with MC38-OVA or EG7 tumor cells. After 10 days, mice were adoptively transferred with naïve CTV-labeled OT-I CD8^+^ T cells and treated, or not, with anti-PD-1 mAb (250 µg, i.v.). **(F)** Experimental setup. **(G–I)** OT-I CD8^+^ T cell proliferation was assessed on day 3 in the draining lymph node. **(G)** Representative histograms showing CTV dilution in OT-I CD8^+^ T cells. **(H)** Quantification of OT-I CD8^+^ T cell replication index in lymph nodes from mice bearing MC38-OVA. Anti-PD-1 mAb-treated mice were compared with PBS- or isotype-injected mice with similar results. Compiled from five independent experiments with 12–19 mice per group. **(I)** Absolute numbers of OT-I CD8^+^ T cells in tumor-draining lymph nodes were assessed 3 days later. Compiled from four independent experiments with a total of 10–12 mice per group. **(J and K)** Quantification of OT-I CD8^+^ T cell (J) replication index and (K) absolute numbers in tumor-draining lymph nodes from mice bearing EG7. Compiled from three independent experiments with a total of seven to nine mice per group. Statistical analyses were performed using *t* tests (E, I, J and K) or two-way ANOVA (H). *, P < 0.05; **, P < 0.01; ***, P < 0.001.

While anti-PD-1 mAb is known to act at the tumor site, recent studies have pointed out an additional activity in the periphery. With the intent to evaluate the contribution of lymph nodes in anti-PD-1 mAb treatment and to study its mechanism therein, we used FTY720 to block T cell egress from secondary lymphoid organs in mice with established MC38-OVA tumors. FTY720 treatment was started 10 days after tumor implantation to ensure that T cell priming had already been initiated (Fig. 1 A). We found that anti-PD-1 mAb treatment exerts a potent antitumor activity in this model. Interestingly, the addition of FTY720 reduced the activity of anti-PD-1 as revealed by lower tumor control and reduced survival (Fig. 1, B and C). These results suggest that T cells present in lymph nodes at the time of anti-PD-1 mAb treatment contribute to the therapeutic benefit.

To get additional insight into the impact of anti-PD-1 mAb treatment on lymph node tumor-specific T cells, we quantified OVA-specific CD8^+^ T cells in draining lymph nodes. PD-1 blockade resulted in a significant increase in the percentage of antigen-specific CD8^+^ T cells (Fig. 1, D and E). To track the early events triggered by PD-1 blockade on CD8^+^ T cells in lymph node, we relied on the adoptive transfer of naïve OT-I CD8^+^ T cells labeled with a proliferation dye at the time of PD-1 injection in tumor-bearing mice (Fig. 1 F). As shown in Fig. 1, G–I, anti-PD-1 mAb treatment increased the absolute number and the proliferation of OT-I CD8^+^ T cells at 3 days after transfer. Similar results were obtained using an additional tumor model (EG7) (Fig. 1, G and J–K).

Together, these data establish that anti-PD-1 mAb acts in the tumor-draining lymph node and promotes the local proliferation of antigen-specific CD8^+^ T cells.

### Anti-PD-1 increases the hallmarks of CD8^+^ T cell activation in draining lymph nodes

**Figure 2.**
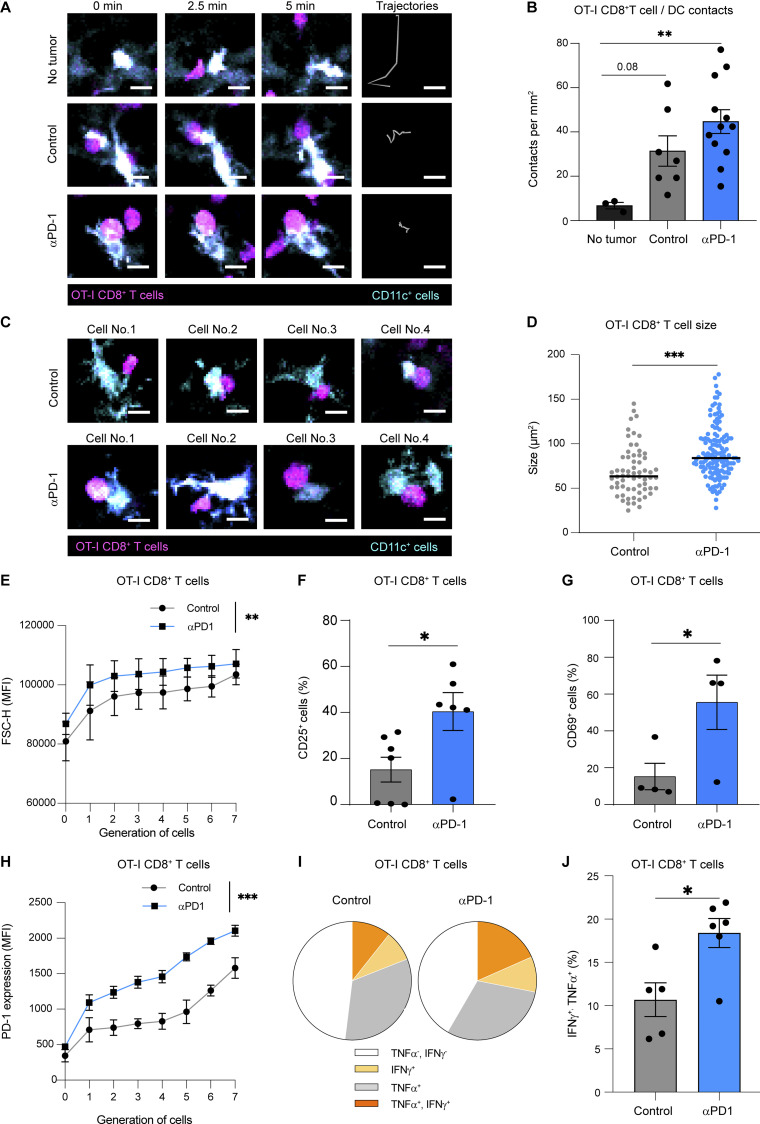
**Anti-PD-1 mAb enhances CD8**^**+**^ **T cell responses in draining lymph nodes. (A–D)** CD11c-eYFP mice were injected s.c. with MC38-OVA tumor cells. After 10 days, mice were adoptively transferred with naïve GFP-expressing OT-I CD8^+^ T cells and treated or not with anti-PD-1 (250 µg, i.v.). Two-photon imaging of tumor-draining lymph nodes was performed on day 2. **(A)** Representative two-photon time-lapse images showing contacts between OT-I CD8^+^ T cells (pseudocolored in magenta) and CD11c reporter positive cells (pseudocolored in cyan) and OT-I CD8^+^ T cell tracks (during 10 min). Scale bar, 10 µm. **(B)** Quantification of the density of stable T cell–DC contacts (lasting >5 min). **(C)** Representative two-photon image of stable T cell–DC contact, illustrating the blastic phenotype seen in anti-PD-1 mAb–treated mice. Scale bar, 10 µm. **(D)** Quantification of the size of OT-I CD8^+^ T cell stably interacting with DCs in the indicated groups. Results (B–D) are compiled from 7 to 12 movies performed in two independent experiments with three mice per group. **(E–H)** C57BL/6 mice were injected s.c. with MC38-OVA. After 10 days, mice were adoptively transferred with CTV-labeled OT-I CD8^+^ T cells and treated or not with anti-PD-1 (250 µg, i.v.). **(E)** OT-I CD8^+^ T cell size was estimated by flow cytometry on day 3 using the FSC-H (forward scatter height) parameter in each cell generation identified by CTV dilution. Representative of four independent experiments with three to four mice per group in each experiment. **(F)** Percentage of CD25^+^ cells among OT-I CD8^+^ T cells on day 1. Compiled from two independent experiments with a total of six mice per group. **(G)** Percentage of CD69^+^ cells among OT-I CD8^+^ T cells on day 1. Representative of two independent experiments with three to four mice per group in each experiment. **(H)** MFI (mean fluorescence intensity) of PD-1 expression on OT-I CD8^+^ T cells was quantified on day 3 for each cell generation. Representative of four independent experiments with three to four mice per group in each experiment. **(I and J)** The production of TNF-α and IFN-γ by OT-I CD8^+^ T cells was measured by intracellular staining after ex vivo restimulation with OVA peptide. **(I)** Pie chart showing cytokine production by OT-I CD8^+^ T cells. **(J)** Quantification of TNF-α^+^IFN-γ^+^ OT-I CD8^+^ T cells. Compiled from two independent experiments with a total of five to six mice per group. Statistical analyses were performed using *t* test (D, F, G, and J) or two-way ANOVA (B, E, and H). *, P < 0.05; **, P < 0.01; ***, P < 0.001.

**Figure S1.**
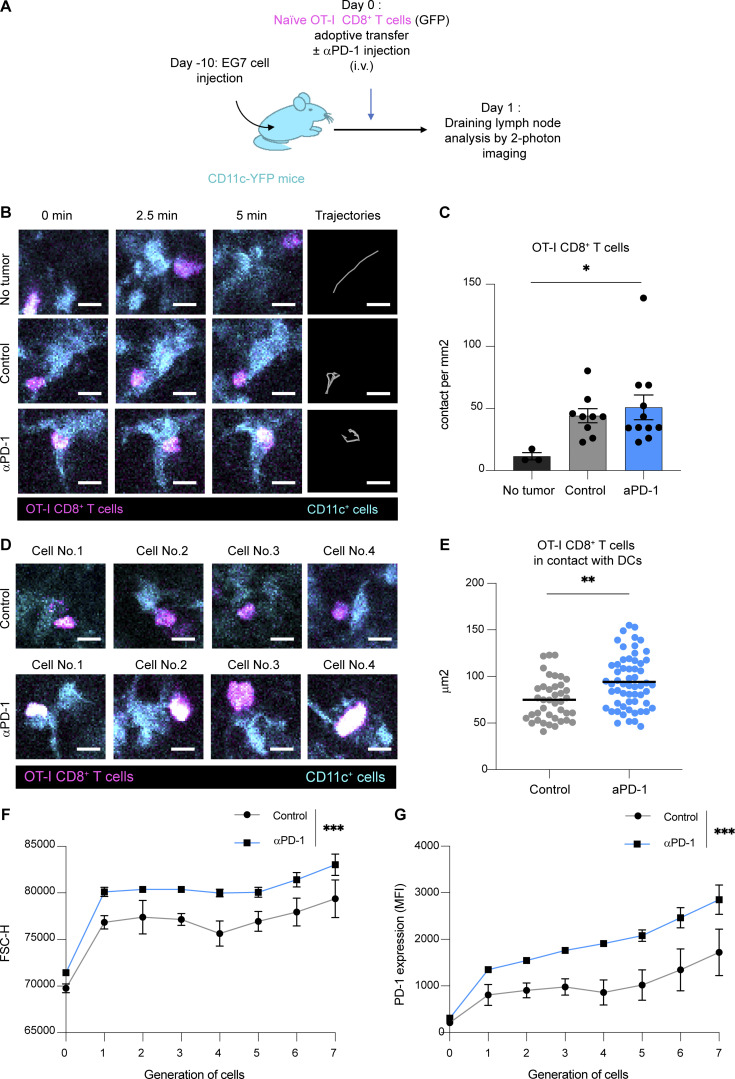
**Anti-PD-1 mAb activity promotes CD8**^**+**^ **T cell responses in the draining lymph node of EG7 tumor–bearing mice. (A–E)** CD11c-eYFP mice were injected s.c. with EG7 tumor cells. After 10 days, mice were adoptively transferred with GFP-expressing OT-I CD8^+^ T cells and treated or not with anti-PD-1 mAb (250 µg, i.v.). Two-photon imaging of tumor-draining lymph nodes was performed on day 1. **(A)** Experimental setup. **(B)** Representative two-photon time-lapse images showing contacts between OT-I CD8^+^ T cells (pseudocolored in magenta) and CD11c reporter positive cells (pseudocolored in cyan) and OT-I CD8^+^ T cell tracks (during 10 min). Scale bar, 10 µm. **(C)** Quantification of the density of stable T cell–DC contacts (lasting >5 min). **(D)** Representative two-photon images of stable T cell–DC contacts, illustrating the blastic phenotype seen in anti-PD-1 mAb–treated mice. Scale bar, 10 µm. **(E)** Quantification of the size of OT-I CD8^+^ T cell stably interacting with DCs in the indicated groups. Results are compiled from 9–11 movies from two independent experiments. **(F and G)** C57BL/6 mice were injected s.c. with EG7 tumor cells. After 10 days, mice were adoptively transferred with CTV-labeled OT-I CD8^+^ T cells and treated or not with anti-PD-1 mAb (250 µg, i.v.). **(F)** OT-I CD8^+^ T cell size was estimated by flow cytometry on day 3 using the FSC-H parameter in each cell generation identified by CTV dilution. Representative of three independent experiments with two to three mice per group in each experiment. **(G)** MFI of PD-1 expression on OT-I CD8^+^ T cells was quantified on day 3 for each cell generation. Representative of three independent experiments with two to three mice per group in each experiment. Statistical analyses were performed using *t* test (E) or two-way ANOVA (C, F, and G). *, P < 0.05; **, P < 0.01; ***, P < 0.001.

**Figure S2.**
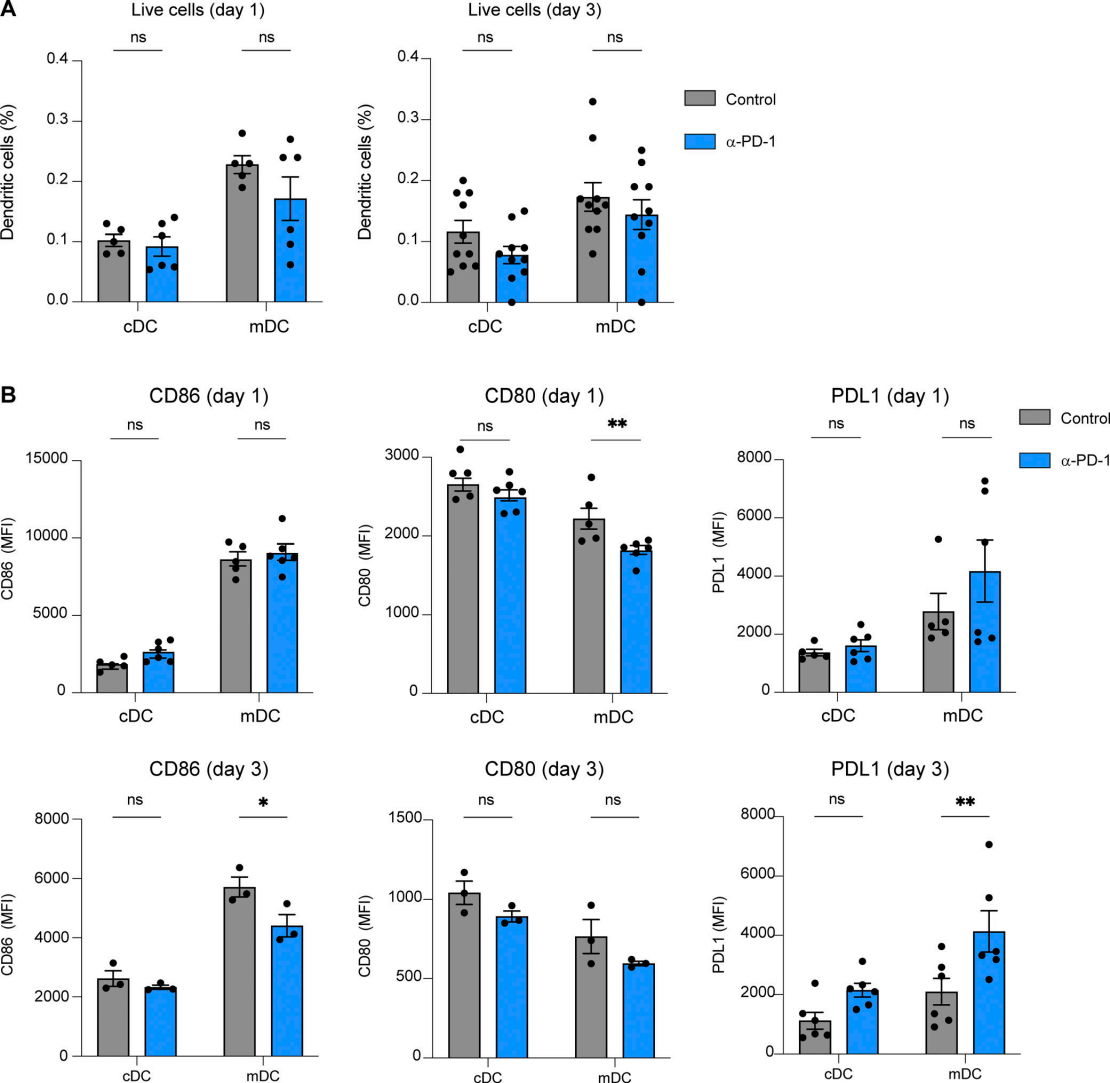
**Anti-PD-1 treatment has little impact on DC phenotype in the tumor-draining lymph node. (A and B)** C57BL/6 mice were injected s.c. with MC38-OVA tumor cells (0.5.10^6^ cells). After 10 days, mice were treated with anti-PD-1 or an isotype control (250 µg, i.v.). On day 1 and day 3, lymph node DC phenotype was assessed by flow cytometry. Frequency (A) and phenotype (B) of migratory DCs (mDC, CD11c^int^, MHC class II^high^) and conventional DC (cDC, CD11c^high^, MHC class II^int^). Representative of two independent experiments. Statistical analyses were performed using two-way ANOVA. *, P < 0.05; **, P < 0.01.

To visualize the early consequences of anti-PD-1 mAb treatment, we analyzed CD8^+^ T cell–DC interactions in entire lymph nodes using two-photon imaging on day 2 after injection. As expected, OT-I CD8^+^ T cells remained highly motile and did not form stable contact with DCs in tumor-free mice. By contrast, a subset of OT-I CD8^+^ T cells was found engaged with DCs in tumor-bearing mice, revealing the presence of antigen-presenting DCs (Fig. 2, A and B; and Video 1). While the frequency of contact did not appear modified by anti-PD-1 mAb, CD8^+^ T cells forming contact with DCs were larger in the presence of anti-PD-1 mAb (Fig. 2, C and D). This blastic phenotype of antigen-specific T cells was also confirmed using flow cytometry (Fig. 2 E). As an additional read-out for T cell activation, we observed a higher frequency of CD69 and CD25 positive OT-I CD8^+^ T cells and a stronger PD-1 upregulation during T cell division in response to anti-PD-1 (Fig. 2, F–H). These effects were reproduced in the EG7 (OVA-expressing EL-4) tumor model (Fig. S1, A–G). Interestingly, we did not observe major changes in DC frequency and activation in the tumor-draining lymph node upon anti-PD-1 treatment, suggesting that additional factors contributed to improved T cell activation (Fig. S2, A and B). Finally, to test whether the enhanced T cell activation phenotype promoted by anti-PD-1 mAb was reflected at the functional level, we analyzed cytokine production (IFN-γ and TNF-α) by OT-I CD8^+^ T cells recovered from draining lymph nodes. PD-1 blockade increased the fraction of double-producers within OT-I CD8^+^ T cells (Fig. 2, I and J). Thus, anti-PD-1 mAb exerts potent effects on the quality of antigen-specific CD8^+^ T cell activation in the tumor-draining lymph node, promoting a blastic phenotype, increasing clonal expansion, and enhancing functional capacity.

**Video 1.** **Tumor-specific CD8**^**+**^ **T cells interact with DCs in the draining lymph node, related to** Fig. 2**.** CD11c-eYFP mice were injected s.c. with MC38-OVA tumor cells. After 10 days, mice were adoptively transferred with naïve GFP-expressing OT-I CD8^+^ T cells and treated or not with anti-PD-1 mAb (250 µg, i.v.). Two-photon imaging of tumor-draining lymph nodes was performed on day 2. The movie shows contacts between OT-I CD8^+^ T cells (pseudocolored in magenta) and CD11c reporter positive cells (pseudocolored in cyan) in tumor-bearing mice but not in tumor-free mouse. Total duration = 29 min.

### Anti-PD-1 mAb rapidly binds to Tfh cells

**Figure 3.**
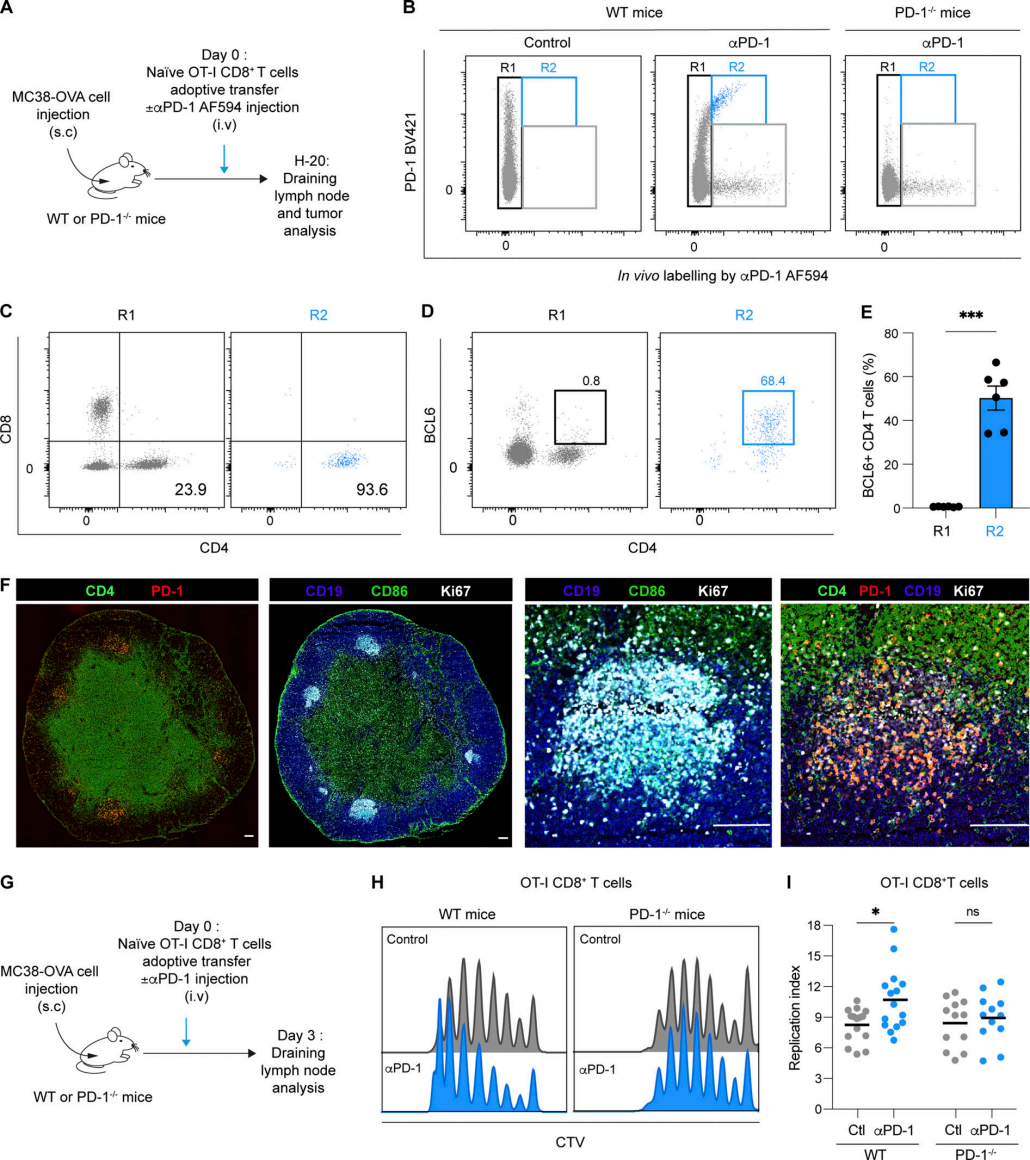
**Anti-PD-1 mAb binds to follicular helper T cells in tumor-draining lymph nodes. (A–E)** C57BL/6 mice were injected s.c. with MC38-OVA tumor cells. After 10 days, tumor-bearing mice were adoptively transferred with naïve GFP-expressing OT-I CD8^+^ T cells and treated with AF594-labeled anti-PD-1 mAb (250 µg, i.v.) or with PBS as a control. PD1^−/−^ mice were used as a control. Flow cytometric analysis of draining lymph nodes was performed 20 h later. **(A)** Experimental setup. **(B)** Representative FACS dot plot showing in vivo labeling by AF594-labeled anti-PD-1 mAb (clone RMP1-14) and ex vivo staining by anti-PD-1 (clone 29F.1A12). Region R1 corresponds to cells that did not exhibit in vivo Ab staining, while R2 corresponds to cells binding the Ab injected in vivo and expressing PD-1 as detected by ex vivo staining. **(C and D)** Expression of CD4 and CD8 markers (C) or CD4 and BCL6 (D) within R1 and R2. **(E)** Quantification of BCL6^+^CD4^+^ T cells within R1 and R2 regions. Representative of two independent experiments with six mice per group in each experiment. **(F)** Immunofluorescence of lymph node sections showing expression of PD-1, CD4, CD19, CD86, and Ki67. Germinal centers (right) are identified using CD19, CD86, and Ki67 markers. Scale bar, 10 µm. Representative of two independent experiments with a total of three mice per group. **(G–I)** C57BL/6 or PD-1^−/−^ mice were injected s.c. with MC38-OVA tumor cells. After 10 days, tumor-bearing mice were adoptively transferred with CTV-labeled OT-I CD8^+^ T cells and treated or not with anti-PD-1 (250 µg, i.v.). Flow cytometric analysis of draining lymph nodes was performed 3 days later. **(G)** Experimental setup. **(H)** Representative histograms showing CTV dilution in OT-I CD8^+^ T cells in WT and PD-1^−/−^ tumor-bearing mice. **(I)** Quantification of OT-I CD8^+^ T cell replication index in lymph nodes of WT and PD-1^−/−^ mice. Compiled from four independent experiments with a total of 12–15 mice per group. Statistical analyses were performed using *t* test (E) or two-way ANOVA (I). ns non-significant; *, P < 0.05; ***, P < 0.001.

**Figure S3.**
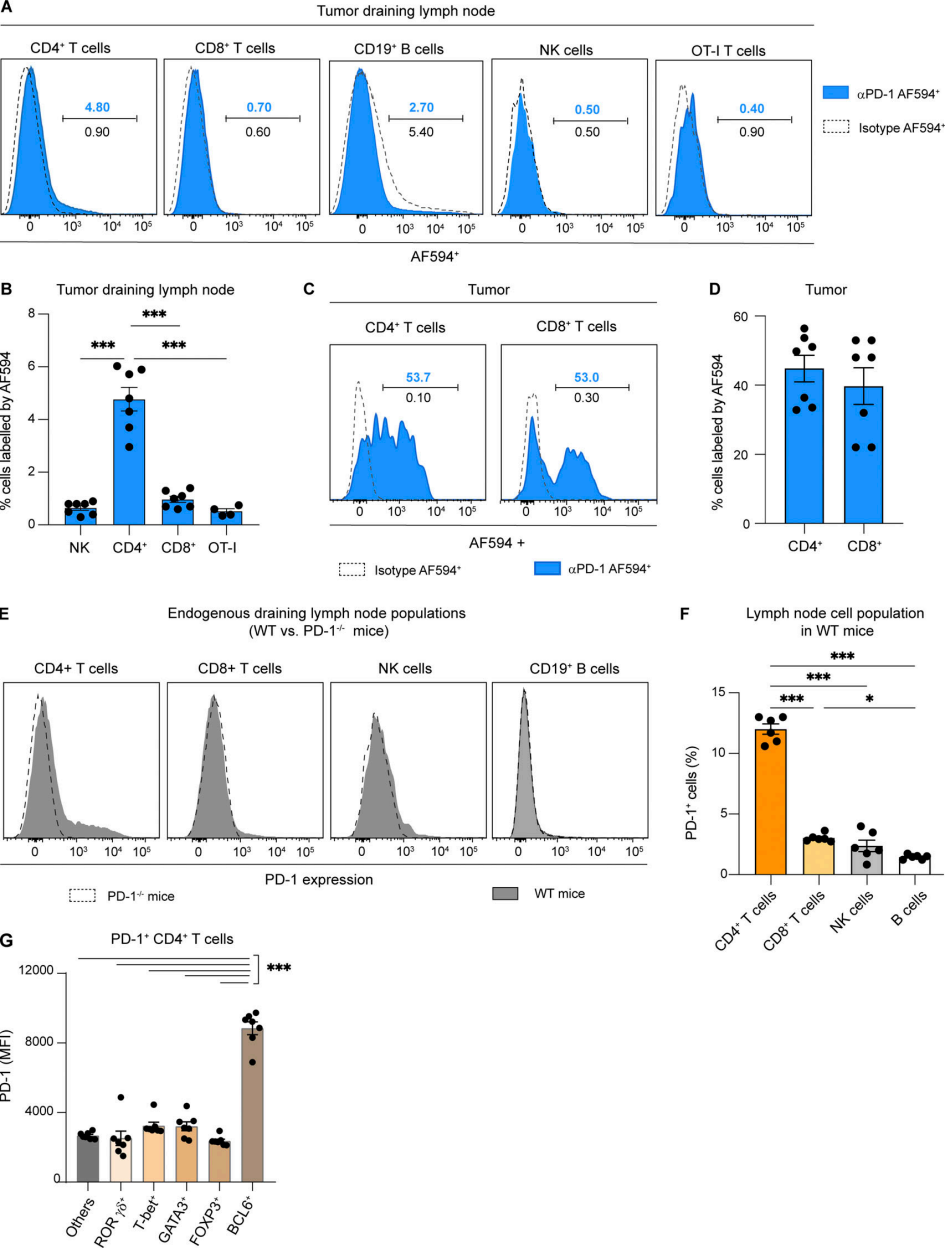
**Anti-PD-1 mAb primarily binds to a subset of CD4**^**+**^ **T cells in tumor-draining lymph nodes. (A–D)** C57BL/6 mice were injected s.c. with MC38-OVA tumor cells. After 10 days, tumor-bearing mice were adoptively transferred with GFP-expressing OT-I CD8^+^ T cells and treated with AF594-labeled anti-PD-Ab or treated with an AF594-labeled IgG2a isotype. Draining lymph node and tumor were analyzed by flow cytometry 20 h later. **(A)** Representative histograms showing in vivo labeling by AF594-labeled anti-PD-1 (percentage shown in blue) or AF594-labeled control isotype (percentage shown in black) on different immune cell subsets in tumor-draining lymph nodes. Note only CD4^+^ T cells specifically bound anti-PD-1 mAb and that a small level (2–5%) of unspecific binding (isotype and anti-PD-1 mAb) is detected on B cells. NK, natural killer. **(B)** Quantification of cell labeling by the AF594-conjugated anti-PD-1 Ab in the indicated subset. **(C)** Representative histograms showing in vivo labeling of tumor-resident CD4^+^ and CD8^+^ T cells by AF594-conjugated anti-PD-1 Ab. **(D)** Quantification of AF594^+^ labeled anti-PD-1 cells in each cell subset. One experiment with five to seven mice per group. **(E–G)** C57BL/6 WT or PD-1^−/−^ mice were injected s.c. with MC38-OVA tumor cells. **(E and F)** PD-1 expression on CD4^+^ or CD8^+^ T cells, CD19^+^ B cells, and NK-1.1^+^ cells was quantified on day 13. Representative histograms (E) and quantification (F) of PD-1^+^–expressing cells in WT mice. Compiled from two independent experiments with a total of six mice per group. **(G)** MFI of PD-1 expressed at the cell surface on different PD-1^+^ CD4^+^ T cell populations, defined by the transcription factors RORγτ, T-bet, GATA3, FoxP3, and BCL6. One experiment with seven mice per group. Statistical analyses were performed using two-way ANOVA, *, P < 0.05; ***, P < 0.001.

We envisioned that anti-PD-1 mAb directly acts on tumor-specific OT-I CD8^+^ T cells in the tumor-draining lymph node where it promotes their expansion. To test whether anti-PD-1 mAb could diffuse in the draining lymph node and identify putative cellular targets, we fluorescently labeled anti-PD-1 mAb before intravenous delivery. To identify unspecific staining, PD-1^−/−^ mice were used as a control (Fig. 3 A). Lymph node cells were stained ex vivo with a different anti-PD-1 mAb clone that did not interfere with the injected mAb (Polesso et al., 2021) to link in vivo labeling with PD-1 surface expression levels. We noted that only the highest PD-1–expressing cells specifically bound the injected Ab, 20 h after injection (Fig. 3 B). Cells binding the injected anti-PD-1 mAb specifically were almost exclusively CD4^+^ T cells with minimal staining on CD8^+^ T cells, including OT-I CD8^+^ T cells (Fig. 3 C and Fig. S3, A and B). By contrast, anti-PD-1 mAb bound a large fraction of CD8^+^ and CD4^+^ T cells at the tumor site (Fig. S3, C and D). Tfh cells have been shown to exhibit a strong expression of PD-1 (Crotty, 2019), and we confirmed that this was the case in tumor-draining lymph nodes (Fig. S3, E–G). We therefore assessed the expression of BCL6, a master regulator of Tfh differentiation and function (Crotty, 2019) within the CD4^+^ T cell binding labeled anti-PD-1 in vivo. Most of these labeled cells were effectively BCL6^+^ (Fig. 3, D and E). Moreover, immunofluorescence of tumor-draining lymph node sections confirmed that the population expressing the highest level of PD-1 were indeed CD4^+^ T cells located in germinal centers (Fig. 3 F). Therefore, Tfh cells represent the major population targeted by the anti-PD-1 mAb in the tumor-draining lymph node.

While our results do not exclude that anti-PD-1 acts on antigen-specific CD8^+^ T cells at later time points when their PD-1 expression may further increase, they suggest that other cells in the lymph node may be the prevailing responders to anti-PD-1 mAb. To test this possibility, we assessed the response of OT-I T cells to anti-PD-1 mAb in PD-1–deficient tumor-bearing hosts (Fig. 3 G). In these settings, there was no effect of anti-PD-1 mAb treatment on OT-I CD8^+^ T cell proliferation (Fig. 3, H and I). Together, these experiments point out Tfh cells as possible contributors to the activity of anti-PD-1 mAb on tumor-specific CD8^+^ T cell proliferation in the draining lymph node.

### Anti-PD-1 mAb acts on mouse and human Tfh cells in vivo

**Figure 4.**
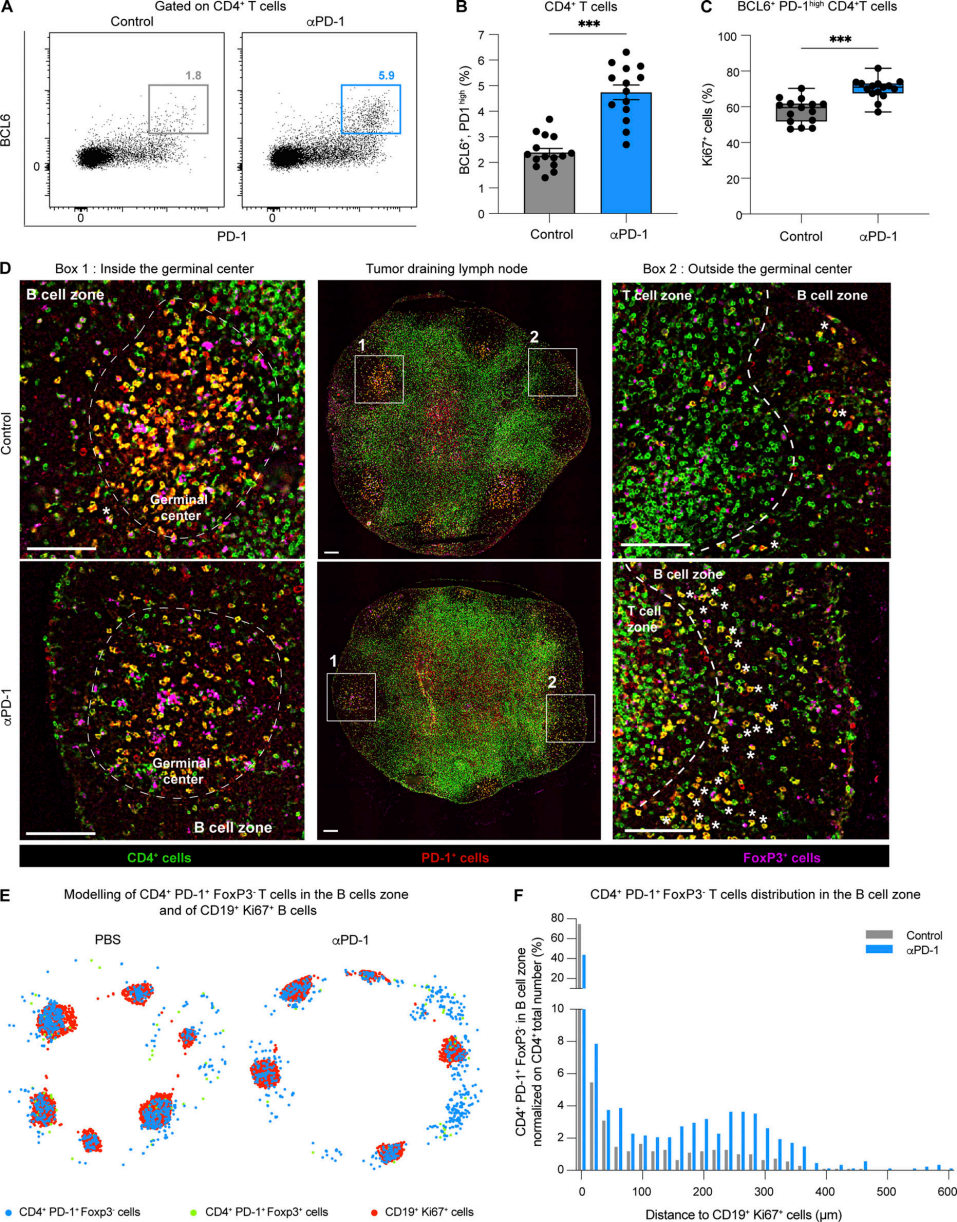
**Tfh cells respond to anti-PD-1 mAb treatment in tumor-draining lymph nodes.** C57BL/6 mice were injected s.c. with MC38-OVA tumor cells. After 10 days, tumor-bearing mice were treated or not with anti-PD-1 mAb (250 µg, i.v.). Draining lymph nodes were analyzed 3 days later by flow cytometry or immunofluorescence. **(A and B)** Representative FACS dot plot (A) and quantification (B) of BCL6^+^ PD-1^high^ cells among CD4^+^ T cells. Compiled from three independent experiments with a total of 15 mice per group. **(C)** Quantification of Ki67^+^ cells among BCL6^+^ PD-1^high^ CD4^+^ T cells. Compiled from three independent experiments with a total of 15 mice per group. **(D)** Lymph node immunofluorescence showing PD-1, CD4, and FoxP3 expression. CD19 and Ki67 staining was included to locate the B cell zone and germinal centers. Region 1 (left) shows a germinal center and region 2 (right) shows an area of the B cell zone without the germinal center. White stars (*) highlight CD4^+^ PD-1^+^ FoxP3^−^ T cells located in the B cell zone but outside germinal centers. Scale bar, 100 µm. Representative of two independent experiments with a total of three mice per group. **(E)** Schematic representation showing CD4^+^ PD-1^+^ FoxP3^−^ T cells in the B cell zone and CD19^+^ Ki67^+^ B cells (to identify germinal centers). **(F)** Distance between individual CD4^+^ PD1^+^ FoxP3^−^ T cells present in the B cell zone and the closest CD19^+^ Ki67^+^ B cells was performed to estimate T cell localization. Representative of two independent experiments with a total of three mice per group. Statistical analyses were performed using *t* test, ***, P < 0.001.

**Figure S4.**
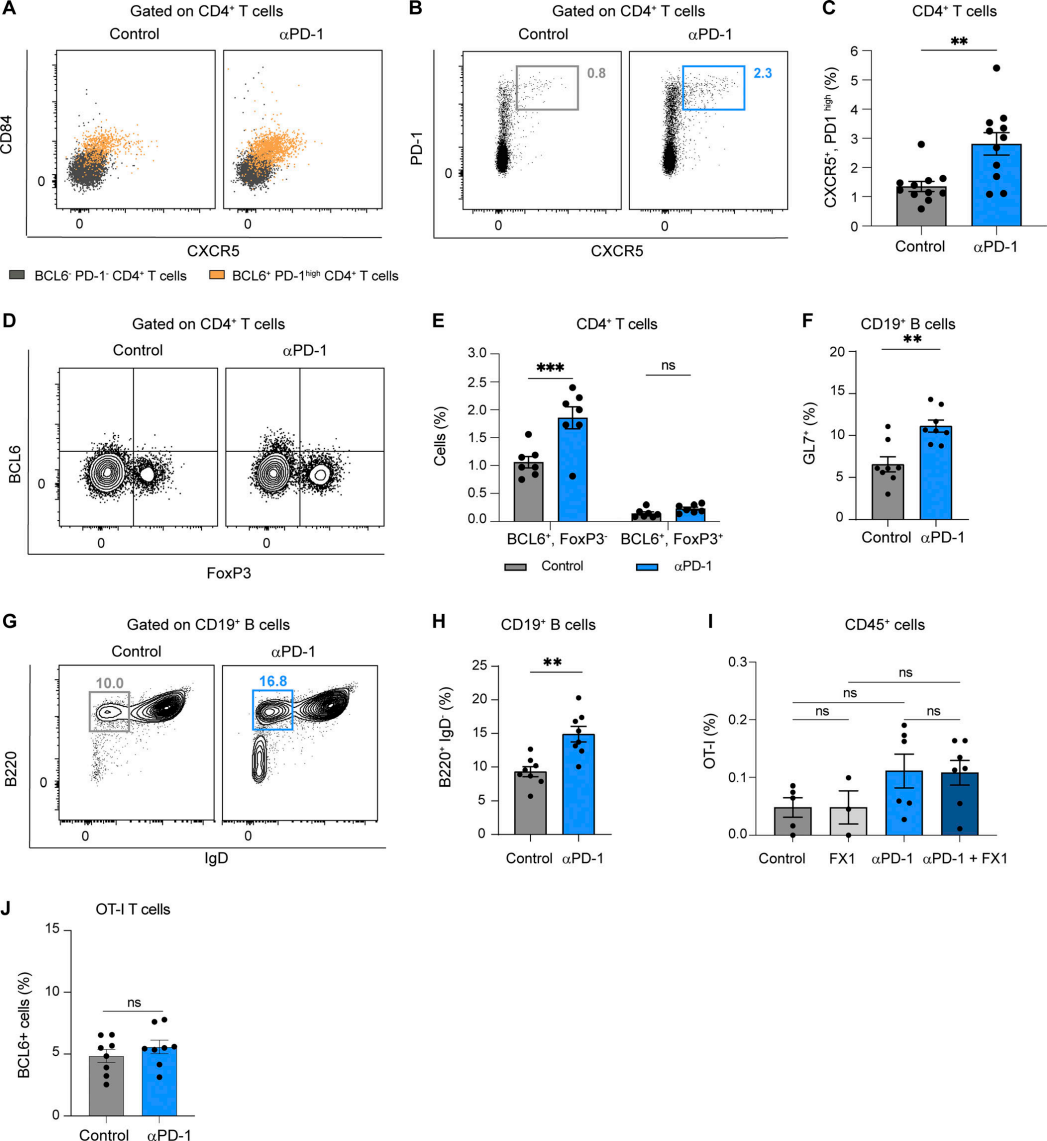
**Impact of anti-PD-1 mAb on lymph node Tfh cells, Tfr cells, B cells, and CD8**^**+**^ **T cells.** C57BL/6 mice were injected s.c. with MC38-OVA tumor cells. After 10 days, tumor-bearing mice were treated or not with anti-PD-1 mAb (250 µg, i.v.). Draining lymph node flow cytometry analysis was performed 3 days later. **(A)** Representative dot plots showing CXCR5 and CD84 expression in CD4^+^ BCL6^+^ PD-1^high^ T cells compared with CD4^+^ BCL6^−^ PD-1^−^ T cells. **(B and C)** (B) Representative FACS dot plots and (C) quantification of CXCR5^+^, PD-1^high^ cells among CD4^+^ T cells. Compiled from three independent experiments with a total of 11 mice per group. **(D and E)** (D) Representative FACS dot plots and (E) quantification of BCL6^+^Foxp3^−^ and BCL6^+^FoxP3^+^ among CD4^+^ T cells (*n* = 7 mice per group). **(F–H)** (F) Quantification of GL7^+^ CD19^+^ B cells. Representative dot plot (G) and quantification (H) of B220^+^ IgD^−^ cells among CD19^+^ B cells. Compiled from two independent experiments with a total of eight mice per group. (**I and J**) C57BL/6 mice were injected s.c. with MC38-OVA tumor cells. After 10 days, mice were adoptively transferred by GFP-expressing OT-I CD8^+^ T cells and treated or not with anti-PD-1 mAb (250 µg, i.v.). **(I)** Tumor-bearing mice received either two doses of the BCL6 inhibitor FX1 or a vehicle as a control. On day 3, OT-I T cells were quantified at the tumor site. **(J)** The small fraction of BCL-6 expressing specific CD8^+^ T cells in the lymph node do not preferentially expand upon anti-PD-1 treatment. Statistical analyses were performed using *t* test (C, F, H, and J), one-way ANOVA (I), or two-way ANOVA (E). ns, non significant; **, P < 0.01, ***, P < 0.001.

Having shown that anti-PD-1 mAb preferentially binds Tfh cells in lymph nodes, we assessed potential changes in these cells (expressing Tfh markers BCL6, PD-1^high^, CXCR5, and CD84) during therapy. As shown in Fig. 4, A and B; and Fig. S4 A, we observed a robust increase in BCL6^+^ PD-1^high^ CD4^+^ T cells upon anti-PD-1 mAb treatment, and most of these cells also expressed CXCR5 and CD84. A similar increase was observed by gating on CD4^+^CXCR5^+^PD-1^high^ T cells (Fig. S4, B and C). We confirmed also that Tfh cells but not Tfr (T follicular regulatory) cells (defined by BCL6, PD-1, and FoxP3) accumulated in response to anti-PD-1 mAb (Fig. S4, D and E). Notably, this increase was associated with an enhanced proliferation of BCL6^+^PD-1^high^ CD4^+^ T cells upon treatment as detected by Ki67 staining (Fig. 4 C). Consistent with an increase in Tfh cell activity mediated by anti-PD-1 mAb, we observed enhanced germinal center B cell responses as revealed by an increased frequency of GL7^+^ and B220^+^IgD^−^ B cells (Fig. S4, F–H) in response to PD-1 blockade.

Of note, high PD-1 expression on Tfh cells has been shown to be essential for their sequestration in the germinal center (Shi et al., 2018). To test whether anti-PD-1 mAb affects localization of Tfh cells, we analyzed the distribution of CD4^+^PD-1^high^ FoxP3^−^ cells present in the B cell zone of the lymph node. As shown in Fig. 4, D–F, anti-PD-1 mAb promoted an accumulation of CD4^+^PD-1^high^ FoxP3^−^ T cells outside of germinal centers. Altogether, our results indicate that mouse Tfh cells respond to anti-PD-1 mAb in lymph nodes.

**Figure 5.**
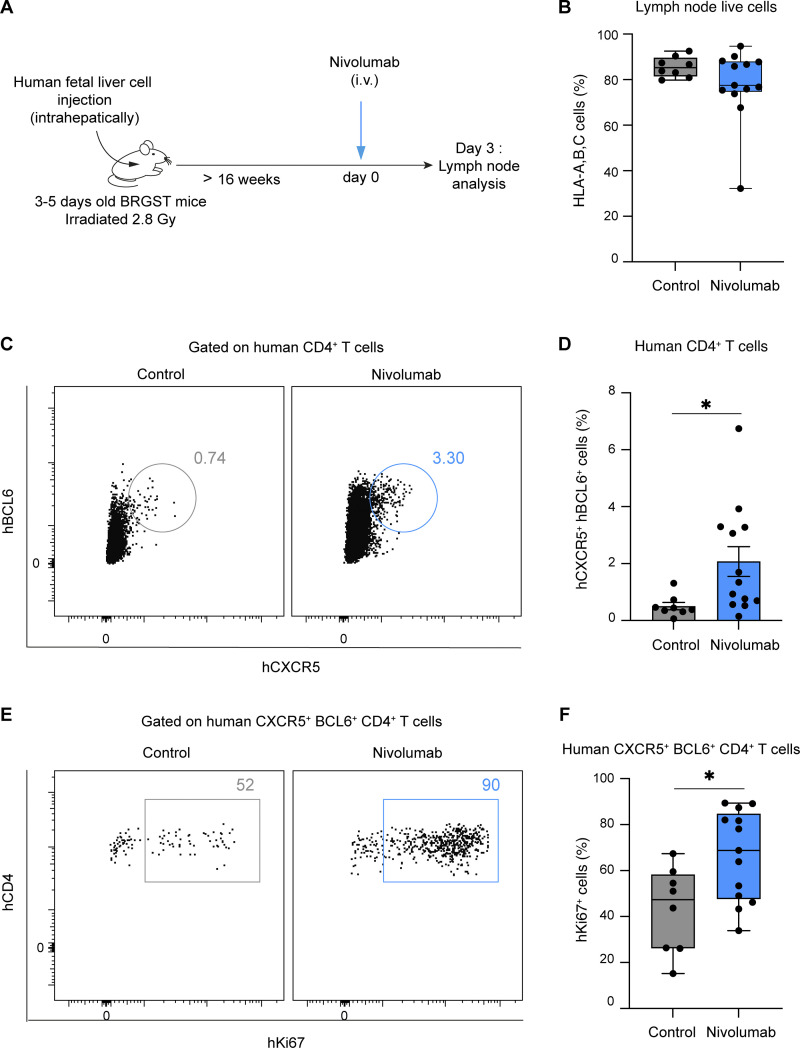
**Human Tfh cells proliferate in response to nivolumab in mice with a humanized immune system. (A–F)** BRGST HIS mice were treated i.v. with 250 µg nivolumab or with PBS and analyzed 3 days later. **(A)** Experimental setup. **(B)** Lymph node reconstitution with human cells was confirmed by HLA-A, B, C staining of live cells. **(C and D)** Representative FACS dot plots (C) and quantification (D) of CD4^+^ BCL6^+^ CXCR5^+^ human T cells in mice treated with nivolumab or PBS. **(E and F)** Representative FACS dot plots (E) and quantification (F) of Ki67^+^ cells among CD4^+^ BCL6^+^ CXCR5^+^ human T cells. Compiled from three independent experiments with a total of 7–13 mice per group and generated from six different human donors. Statistical analyses were performed using *t* test (B, D, and F). *, P < 0.05.

To extend these findings in a humanized setting, we took advantage of BRGST (BALB/c Rag2^−/−^ IL2rg^−/−^ SirpaNOD TSLP) Human Immune system mice in which lymph nodes develop robustly due to the expression of TLSP (thymic stromal lymphopoietin). These lymph nodes contain functional human B and T cells including Tfh cells (Li et al., 2018), offering the possibility to assess the in vivo consequences of anti-PD-1 mAb (nivolumab) treatment on human T cells in an organized secondary lymphoid tissue (Fig. 5, A and B). As shown in Fig. 5, C–F, injection of nivolumab resulted in a substantial increase in human Tfh (CXCR5^+^BCL6^+^CD4^+^ T cells) cell frequency and proliferation. These results suggest that nivolumab also acts on human Tfh cells in vivo.

### Tfh cells play a key role in CD8^+^ T cell response to PD-1 blockade

**Figure 6.**
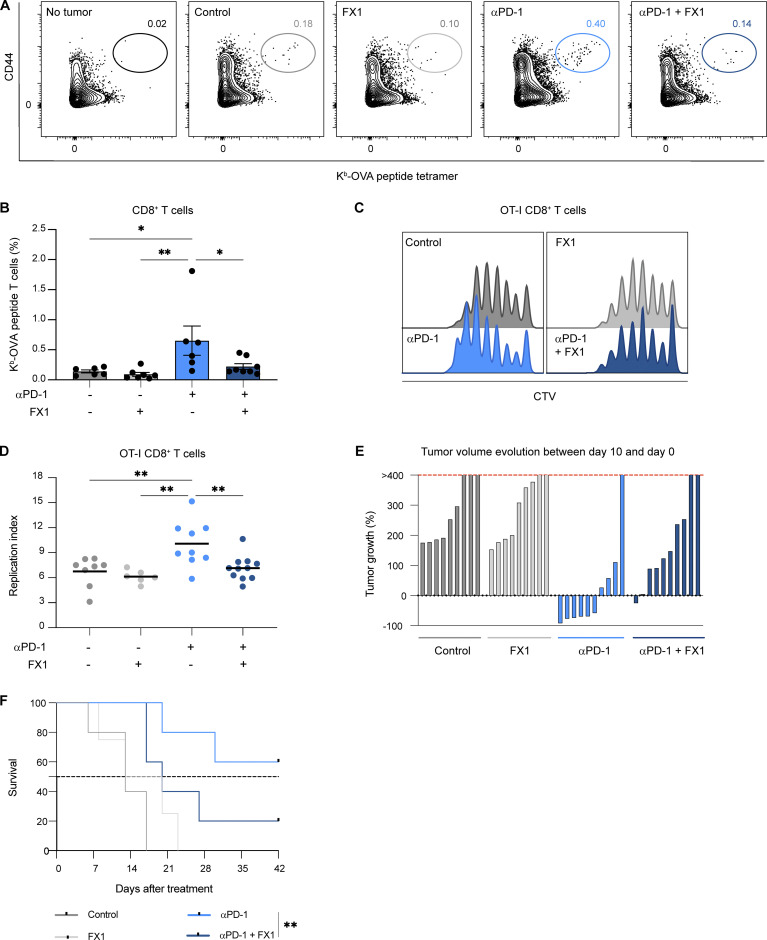
**Anti-PD-1 mAb activity in the draining lymph node is BCL6 dependent. (A and B)** C57BL/6 mice were injected s.c. with MC38-OVA. After 10 days, mice were treated or not with anti-PD-1 mAb (250 µg, i.v.) and received either three doses of the BCL6 inhibitor FX1 or a vehicle as a control. Representative FACS dot plots (A) and quantification (B) of H2-K^b^-OVAp tetramers^+^ CD8^+^ T cells 5 days after treatment. Compiled from two independent experiments with five to eight mice per group. **(C and D)** C57BL/6 mice were injected s.c. with MC38-OVA. After 10 days, mice were adoptively transferred by CTV-labeled OT-I CD8^+^ T cells and treated or not with anti-PD-1 mAb (250 µg, i.v.) and received either two doses of the BCL6 inhibitor FX1 or a vehicle as a control. OT-I CD8^+^ T cell proliferation was assessed 3 days later. **(C)** Representative histograms showing CTV dilution in OT-I CD8^+^ T cells. **(D)** Quantification of OT-I CD8^+^ T cell replication index in lymph nodes. Compiled from two independent experiments with 6–11 mice per group. **(E and F)** C57BL/6 mice were injected s.c. with MC38-OVA. After 10 days, mice were treated or not with anti-PD-1 mAb (250 µg, i.v.). Some mice were injected every 2 days with FX1 or vehicle until day 10. **(E)** Evolution of tumor volume between day 0 and day 10. **(F)** Survival curves for the indicated groups. The dashed line corresponds to 50% survival. Compiled from two independent experiments with a total of 9–10 mice per group. Statistical analyses were performed using two-way ANOVA (B and D) or a log-rank test (F). *, P < 0.05; **, P < 0.01.

We next assessed the functional impact of Tfh cells on tumor-specific CD8^+^ T cell response to anti-PD-1 mAb by targeting the BCL6 transcription factor. To this end, we used the BCL6 inhibitor FX-1 that binds the BTB domain of BCL6, thus preventing BCL6 transcriptional activity (Cai et al., 2020, 2021; Cardenas et al., 2016). FX-1 treatment alone had no effect on endogenous tumor-specific CD8^+^ T cell responses. However, treatment of mice with FX1 prevented anti-PD1 mAb-induced boosting of antigen-specific CD8^+^T cells in the draining lymph node (Fig. 6, A and B). Similarly, FX-1 impaired the impact of anti-PD-1 mAb on OT-I CD8^+^ T cell proliferation in the lymph node (Fig. 6, C and D) but minimally affected OT-I CD8^+^ T cells at the tumor site (Fig. S4 I). Finally, FX-1 treatment reduced tumor control and the survival of mice treated with anti-PD-1 mAb (Fig. 6, E and F). It is unlikely that FX-1 acted directly on BCL6-expressing specific CD8^+^ T cells as this population represented <5% and was not increased by anti-PD-1 treatment (Fig. S4 J). Our results suggest that BCL6-dependent cells, most likely Tfh cells, are major contributors to PD-1 blockade activity in the draining lymph node.

### IL-4 is essential for anti-PD-1–mediated enhancement of tumor-specific CD8^+^ T cell responses

**Figure 7.**
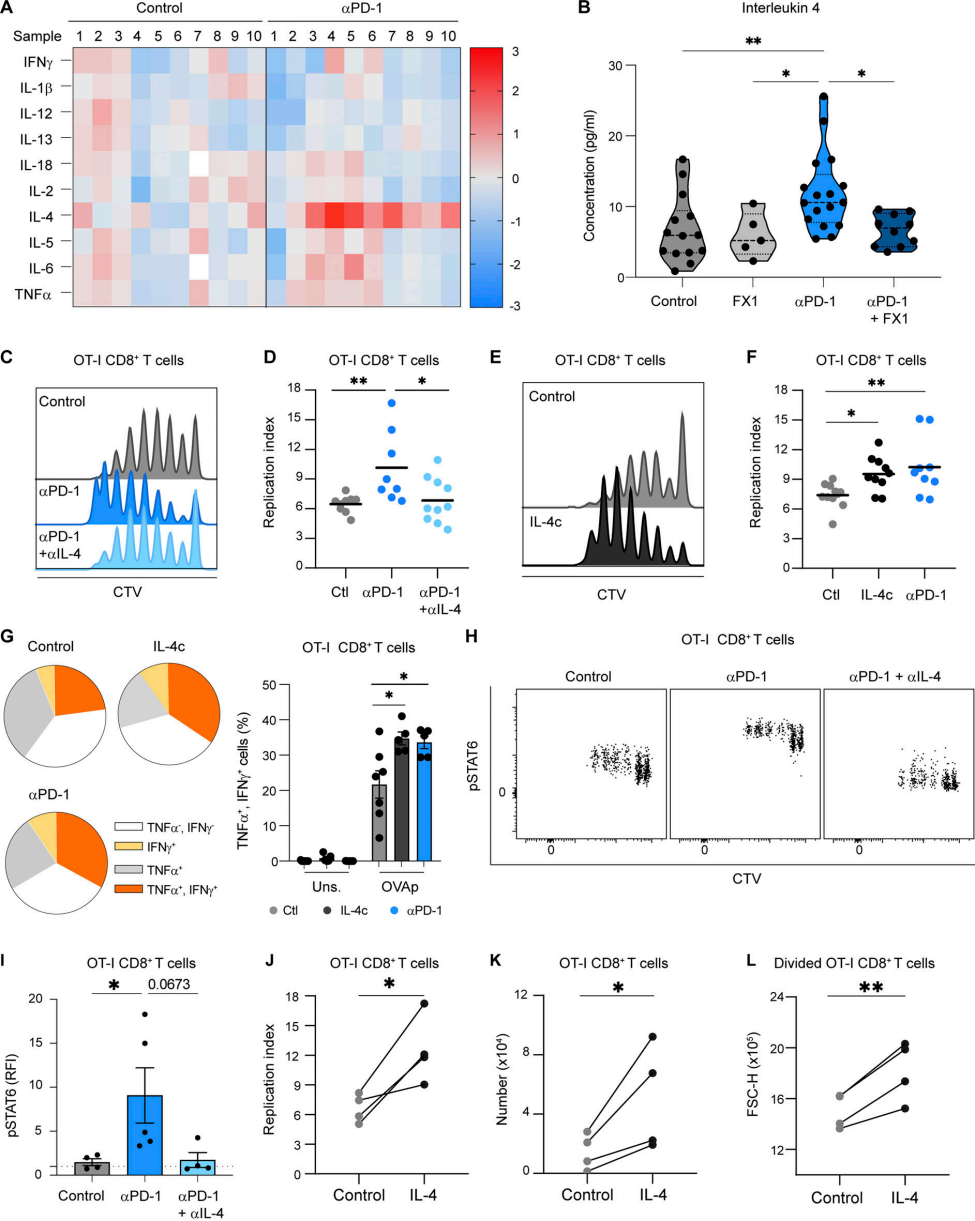
**IL-4 is essential for anti-PD-1–mediated enhancement of tumor-specific CD8**^**+**^ **T cell responses. (A)** C57BL/6 mice were injected s.c. with MC38-OVA tumor cells. After 10 days, mice were treated or not with anti-PD-1 mAb (250 µg, i.v.). Heat map showing the cytokine landscape (as detected by multiplex protein assay) of the draining lymph nodes, 3 days after anti-PD-1 mAb treatment. Each value was normalized to the mean value of control samples (untreated). Compiled from three independent experiments with a total of 10 mice per group. **(B)** Tumor-bearing mice were treated or not with anti-PD-1 mAb (250 µg, i.v.) and received either two doses of the BCL6 inhibitor FX1 or a vehicle as a control. IL-4 concentration was measured in the draining lymph node 3 days after treatment. Compiled from two independent experiments with a total of at least five mice per group. **(C and D)** Tumor-bearing mice were transferred with CTV-labeled OT-I CD8^+^ T cells and treated with anti-PD-1 Ab or left untreated and received either two doses of anti-IL-4 Ab or an isotype control. **(C)** Representative histograms showing CTV dilution in OT-I CD8^+^ T cells in the indicated group. **(D)** Quantification of OT-I CD8^+^ T cell replication index. Compiled from three independent experiments with a total of 9–10 mice per group. **(E–G)** Tumor-bearing mice were transferred with CTV-labeled OT-I CD8^+^ T cells and mice were treated (or not) with anti-PD-1 mAb (250 µg, i.v.) or received one dose of IL-4 complexes. **(E)** Representative histograms showing CTV dilution in OT-I CD8^+^ T cells. **(F)** Quantification of OT-I CD8^+^ T cell replication index. Compiled from four independent experiments with a total of 10–12 mice per group. **(G)** Pie charts and quantification showing TNF-α and IFN-γ production in OT-I CD8^+^ T cells. Compiled from two independent experiments with a total of five to six mice per group. **(H and I)** C57BL/6 mice were injected s.c. with MC38-OVA tumor cells. After 10 days mice were treated with anti-PD-1 mAb with or without anti-IL4 mAb. Control mice received the appropriate isotype controls (250 µg, i.v.). Detection of p-STAT6 in OT-I CD8^+^ T cells 2 days after treatment. RFI, ratio of fluorescence intensity. **(H)** Representative dot plots showing p-STAT6 staining in each cell generation identified by CTV dilution. **(I)** MFI of p-STAT6 staining on OT-I CD8^+^ T cells normalized to that measured with an isotype control. Representative of two independent experiments with four to five mice per group in each experiment. **(J–L)** OT-I CD8^+^ T cells were labeled by CTV and in vitro activated with OVA peptide. IL-4 was added on day 1 and 2 and CTV dilution was assessed on day 3. **(J)** Quantification of OT-I CD8^+^ T cell replication index in the presence or absence of IL-4. **(K)** Absolute number of OT-I CD8^+^ T cells in the presence or absence of IL-4. **(L)** The average size of divided OT-I CD8^+^ T cells was estimated by flow cytometry. Results from four independent experiments are shown. Each dot represents the mean of at least three replicates measured in each experiment. Statistical analyses were performed using two-way ANOVA (B, D, F, and I) and paired *t* test (J–L). *, P < 0.05; **, P < 0.01.

**Figure S5.**
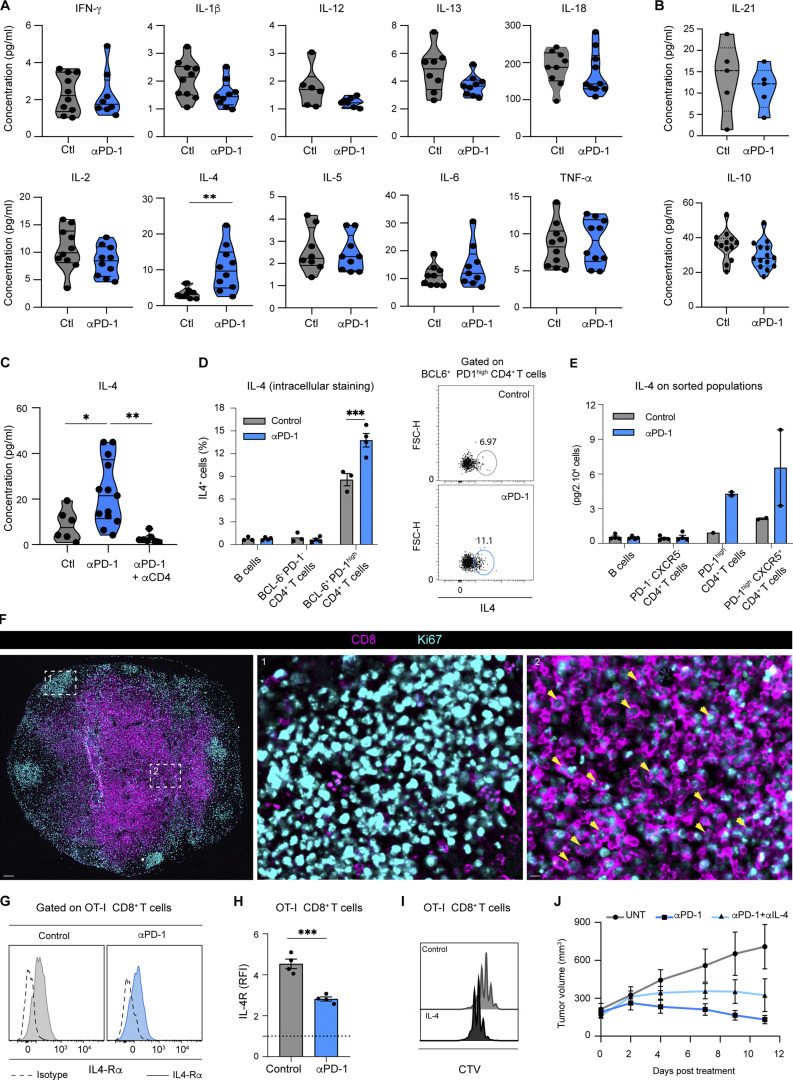
**Role of IL-4 during response to anti-PD-1 mAb. (A and B)** Cytokine landscape of tumor-draining lymph nodes 3 days after anti-PD-1 mAb treatment. Tested cytokines included (A) IFN-γ, IL-1β, IL-12, IL-13, IL-18, IL-2, IL-4, IL-5, IL-6, TNF-α, and (B) IL-21 and IL-10. The calculated concentrations correspond to 1 × 10^6^ lysed lymph node cells resuspended in 50 μl of lysis buffer. Compiled from three independent experiments with 10 mice per group. **(C)** C57BL/6 mice were injected s.c. with MC38-OVA. After 10 days, mice were treated or not with anti-PD-1 mAb (250 µg, i.v.) and received i.p. either three doses of an anti-CD4 (200 µg) or PBS as a control. IL-4 production in tumor-draining lymph node was assessed 3 days after treatment. Compiled from two independent experiments with 7–13 mice per group. **(D)** Quantification of IL-4^+^ cells by intracellular staining in different CD4^+^ T cells subset or in CD19^+^ B cells 3 days after anti-PD-1 mAb treatment. **(E)** The indicated cell populations were sorted from tumor-draining lymph node 3 days after anti-PD-1 mAb treatment. IL-4 concentrations shown correspond to 2 × 10^4^ sorted cells resuspended in 50 μl. Each dot represents one sorted population. **(F)** Lymph node immunofluorescence of tumor-bearing mice treated with anti-PD-1 mAb showing CD8 and Ki67 expression. Region 1 (middle image) shows a germinal center and region 2 (right image) shows an area of the T cell zone. Yellow arrows highlight CD8^+^ Ki67^+^ T cells. Scale bar, 100 µm. **(G and H)** (G) Representative histograms and (H) quantification of IL4-Rα expression on OT-I CD8^+^ T cells normalized to that measured with an isotype control. **(I)** Representative histograms showing CTV dilution in OT-I CD8^+^ T cells stimulated using OVA peptide in the presence or absence of IL-4 in vitro. **(J)** Tumor-bearing mice were adoptively transferred with OT-I CD8^+^ T cells, treated or not with anti-PD-1 mAb either alone or combined with anti-IL-4 mAb (given every 2 days). Tumor volume was monitored for 10 days. Statistical analyses were performed using *t* test (A, B, D, and H) or one-way ANOVA (C). *, P < 0.05; **, P < 0.01; ***, P < 0.001.

To clarify how Tfh cells may promote CD8^+^ T cell responses during anti-PD-1 mAb treatment, we analyzed the cytokine landscape in the tumor-draining lymph node. Among the 12 cytokines tested, we observed that IL-4 was strongly and specifically upregulated in PD-1–treated animals in contrast to all other tested cytokines including IL-21 and IL-10 (Fig. 7 A and Fig. S5, A and B). As IL-4 is a well-known Tfh cell–associated cytokine, we tested whether Tfh cells are indeed responsible for the observed increase in IL-4 levels during PD-1 blockade. We noted that CD4^+^ T cell depletion strongly impaired IL-4 production (Fig. S5 C). Most importantly, FX-1 treatment abrogated the increase in IL-4 levels in mice treated with anti-PD-1 (Fig. 7 B). The major role of Tfh cells in IL-4 production induced by PD-1 blockade was also revealed by intracellular cytokine staining and by assaying cytokine levels on sorted immune cell populations (Fig. S5, D and E). Together, these data suggest that Tfh cells are essential to the increase of IL-4 levels in lymph nodes following anti-PD-1 mAb treatment.

To test the importance of IL-4 in anti-PD-1 mAb activity in the lymph node, we treated tumor-bearing mice with anti-IL4 mAb in the context of anti-PD-1 mAb treatment. We observed that anti-IL-4 mAb strongly impaired the activity of anti-PD-1 mAb in antigen-specific CD8^+^ T cell proliferation (Fig. 7, C and D). Conversely, treating tumor-bearing mice with IL-4 complexes (IL-4 bound to anti-IL-4 mAb to enhance its in vivo half-life) recapitulated the effect of anti-PD-1 mAb on OT-I CD8^+^ T cell expansion (Fig. 7, E and F) and TNF-α^+^IFN-γ^+^ double-producer T cells (Fig. 7 G). Thus, IL-4 appeared both necessary and sufficient for the effects of PD-1 blockade on CD8^+^ T cell proliferation and function in the lymph node. To test whether CD8^+^ T cells were sensing IL-4 during anti-PD-1 mAb treatment, we assessed the expression of IL-4R and p-STAT6. As shown in Fig. 7, H and I, p-STAT6 was substantially increased on OT-I CD8^+^ T cells in mice treated with anti-PD-1 mAb, suggesting increased IL-4 signaling. Moreover, p-STAT6 upregulation upon anti-PD1 therapy was abolished by anti-IL-4 Ab treatment (Fig. 7, H and I). The fact that most CD8^+^ T cells upregulated p-STAT6 is consistent with its ability of IL-4 to diffuse throughout the lymph node (Perona-Wright et al., 2010) and with broad distribution of proliferating CD8^+^ T cells seen upon anti-PD-1 mAb (Fig. S5 F). IL-4R was detected on proliferating OT-I CD8^+^ T cells with the levels being lower in anti-PD-1–treated mice (Fig. S5, G and H), possibly due to receptor internalization (Friedrich et al., 1999; Kurgonaite et al., 2015).

To confirm that IL-4 could directly act on CD8^+^ T cell responses, we stimulated OT-I CD8^+^ T cells in vitro in the presence or absence of IL-4. In the presence of IL-4, OT-I CD8^+^ T cells displayed increased size and enhanced proliferative capacity, reminiscent of our in vivo observations in anti-PD-1–treated mice (Fig. 7, J–L; and Fig. S5 I). Of note, anti-IL-4 mAb also reduced the activity of anti-PD-1 on tumor growth as observed with FTY720 or FX-1 treatment, a finding compatible with a partial role of peripheral T cells during anti-PD-1 therapy (Fig. S5 J). In sum, our results suggest that Tfh cell–derived IL-4 promotes antigen-specific CD8^+^ T cell responses in tumor-draining lymph nodes during anti-PD-1 therapy.

### IL-4 is essential for anti-PD-1–mediated enhancement of vaccine-specific CD8^+^ T cell responses

**Figure 8.**
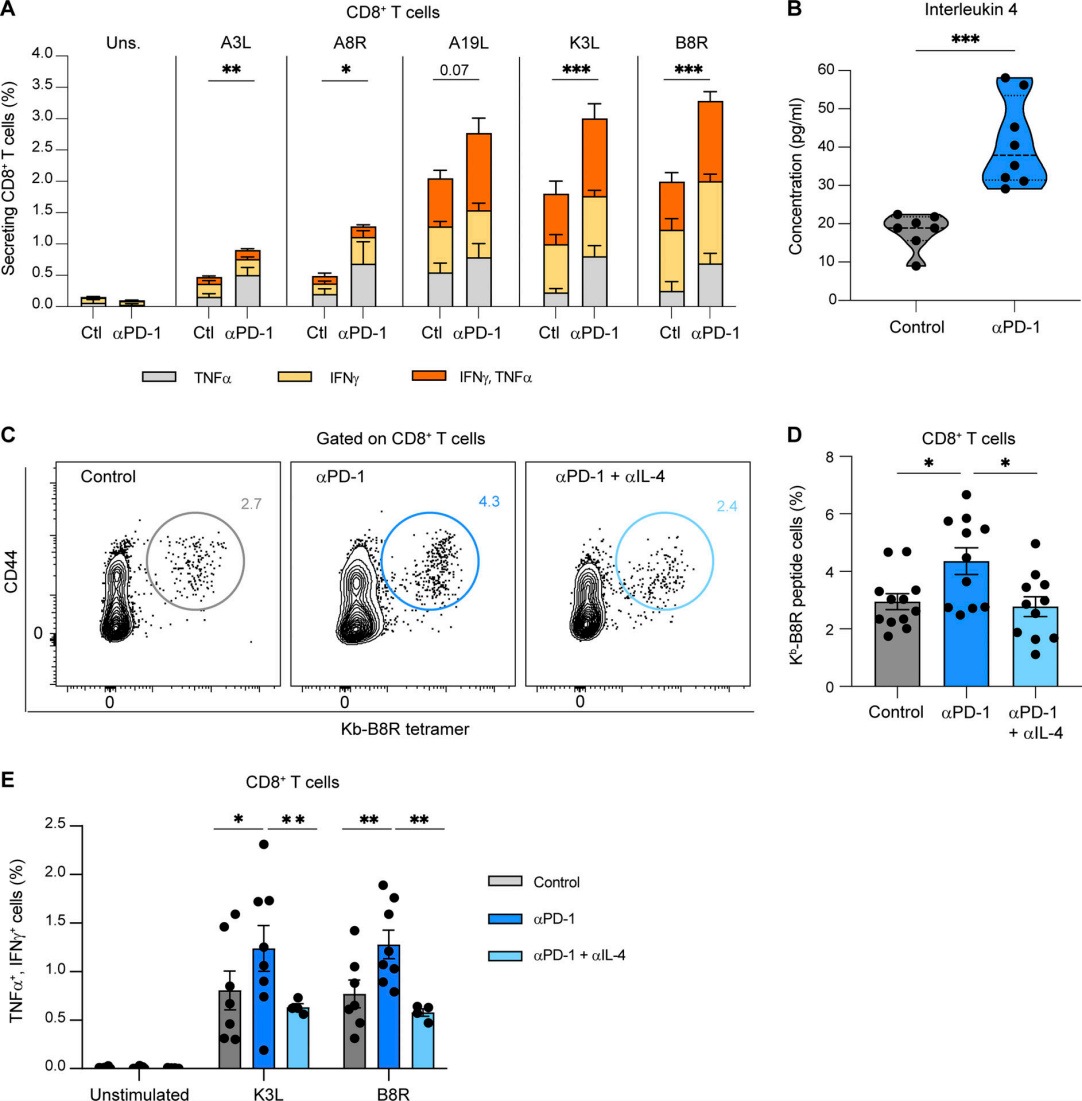
**IL-4 is essential for anti-PD-1–mediated enhancement of vaccine-specific CD8**^**+**^ **T cell responses. (A and B)** C57BL/6 mice were injected into the footpad with 2 × 10^6^ p.f.u. of MVA-HIV-B. After 3 days, mice were treated i.v. with anti-PD-1 or a control isotype. The draining lymph nodes were analyzed on day 6. **(A)** Production of TNF-α and IFN-γ by CD8^+^ T cells after ex vivo stimulation by A3L, A8R, A19L, K3L, or B8R peptides, five known H-2^b^–restricted MVA epitopes. Compiled from two independent experiments with seven to eight mice per group. Uns., unstimulated. **(B)** IL-4 production measured in the lysate of the lymph node draining the site of MVA injection. Compiled from two independent experiments with seven to eight mice per group. **(C–E)** C57BL/6 mice were injected into the footpad with 2 × 10^6^ p.f.u. of MVA-HIV-B. After 3 days, mice were either untreated or treated i.v. with anti-PD-1 Ab combined with two injections of anti-IL-4 mAb or with anti-PD-1 with two injections of an isotype control. Representative FACS contour plots (C) and quantification (D) of H2-K^b^-B8R tetramers^+^ CD8^+^ T cells. Compiled from three independent experiments with 11–12 mice per group. **(E)** Quantification of TNFα^+^ and IFNγ^+^ double-producing CD8^+^ T cells after ex vivo stimulation by K3L or B8R peptides (*n* = 4–9 mice per group). Statistical analyses were performed using two-way ANOVA (A, D, and E) and *t* test (B). *, P < 0.05; **, P < 0.01; ***, P < 0.001.

We next tested whether the mechanism described in tumor-draining lymph nodes induced by anti-PD-1 mAb treatment pertains to other types of CD8^+^ T cell responses and thus sought to analyze T cell responses in a vaccine setting. Mice were injected with a modified vaccinia virus Ankara (MVA) and treated with anti-PD-1 mAb on day 3. We analyzed the CD8^+^ T cell responses against five known epitopes (including the B8R immunodominant epitope) in the draining lymph node using intracellular cytokine staining. For all epitopes, anti-PD-1 increased both the frequency of responding cells and the fraction of IFN-γ^+^-TNF-α^+^ double-producers, revealing, in particular, cryptic responses (Fig. 8 A). Thus, anti-PD-1 can enhance and broaden vaccine responses. As observed in the tumor context, PD-1 blockade induced the production of IL-4 in the draining lymph node (Fig. 8 B). In vivo neutralization of IL-4 interfered with anti-PD-1 activity on vaccine-specific CD8^+^ T cell responses, decreasing the magnitude of CD8^+^ T cell responses as well as the frequency of IFN-γ^+^TNF-α^+^ double-producer T cells (Fig. 8, C–E). Our results suggest that the described mechanism of anti-PD-1 mAb activity in tumor-draining lymph nodes also operates during vaccination.

## Discussion

Here, we describe a new mechanism by which anti-PD-1 mAb promotes antigen-specific CD8^+^ T cell responses by stimulating Tfh cells and IL-4 production in tumor-draining lymph nodes.

Recent studies have shown that anti-PD-1 mAb treatment in cutaneous carcinoma and melanoma patients resulted in the emergence of new TCR clonotypes in the TME, suggesting de novo priming of tumor-reactive T cells in the periphery (Liu et al., 2022; Nagasaki et al., 2022; Wu et al., 2020; Yost et al., 2019). In this respect, the tumor-draining lymph node represents a reservoir for progenitor-exhausted CD8^+^ T cells (Prokhnevska et al., 2023; Rahim et al., 2023) that could recirculate to favor the intratumoral accumulation of newly stimulated T cell clones (Li et al., 2022).

Our work provides direct evidence that anti-PD-1 mAb enhances the priming of antigen-specific T cells acting both on the magnitude and quality of T cell activation in the draining lymph node. Moreover, and in line with recent studies (Fransen et al., 2018; Nagasaki et al., 2022), the egress of T cells generated after anti-PD-1 mAb injection appears to play a partial role in tumor control. Therefore, anti-PD-1 mAb may exert dual effects at the tumor site and in lymphoid organs that likely both contribute to the therapeutic benefit of the treatment.

Understanding whether the mechanisms promoting cytotoxic T lymphocyte responses in the tumor or lymph nodes are similar or different is key. An interesting difference could be the cellular population bound by anti-PD-1 mAb. In this respect, we observed a strong binding to intratumoral T cells (most likely due to the large fraction of exhausted T cells), while limited binding was detected on CD8^+^ T cells in lymph node. Instead, we noted a robust mAb binding on a subpopulation of CD4^+^ T cells that we identified as Tfh cells, suggesting that anti-PD-1 mAb may not act directly on CD8^+^ T cells to promote their activation. Consistently, the use of PD- 1^−/−^ hosts confirmed the importance of PD-1 expression on cells distinct from tumor-specific CD8^+^ T cells during anti-PD-1 mAb therapy.

Several pieces of evidence suggest a major role for Tfh cells in the lymph node response to anti-PD-1 mAb. First, Tfh cells exhibit the highest levels of PD-1 expression in lymph nodes, which likely favors their capture of anti-PD-1 mAb in this location, where antibody diffusion is known to be limited (Arlauckas et al., 2017). Second, we found that anti-PD-1 mAb treatment favors the redistribution of Tfh cells outside of germinal centers, a finding consistent with the fact that high PD-1 expression is essential to confine Tfh cells in the germinal center (Shi et al., 2018). Third, we observed a rapid increase in the frequency of Tfh cells (at least partly due to enhanced proliferation) following anti-PD-1 mAb treatment. Notably, this phenomenon was recapitulated in mice with a humanized immune system treated with nivolumab. Finally, pharmacological inhibition of BCL6, a master regulator of Tfh differentiation and function, impaired anti-PD-1 mAb activity on CD8^+^ T cell responses. Of note, BCL6 can be expressed by other cell types in lymph nodes, including Tfr cells and B cells. We do not favor a direct role for these populations as B cells do not express PD-1, and Tfr cells were poorly impacted by anti-PD-1 mAb.

Consistent with an enhanced Tfh cell activity, we noted increased B cell class switching upon PD-1 blockade. While the present study is focused on the early consequences of anti-PD-1 mAb on lymph node CD8^+^ T cell responses, it will be important to investigate the long-term impact of the described mechanism on antibody responses.

The involvement of Tfh cells in response to anti-PD-1 mAb in lymph nodes represents an intriguing parallel to some recent observations made at the tumor site (Cohen et al., 2022; Gu-Trantien et al., 2013; Magen et al., 2022, *Preprint*; Niogret et al., 2021; Sánchez-Alonso et al., 2020). Indeed, the presence of intratumor CD4^+^ T cells expressing Tfh markers (including CXCL13 and PD-1^high^) in multiple types of carcinomas is associated with better cancer outcomes (Gu-Trantien et al., 2013; Niogret et al., 2021) and favorable responses to anti-PD-1 therapy (Magen et al., 2022, *Preprint*). Of note, these cells were found to interact with intratumoral DCs (Cohen et al., 2022; Magen et al., 2022, *Preprint*). In addition, adoptive transfer of in vitro–generated Tfh-like cells can enhance antitumor activity in mouse models (Cohen et al., 2022; Niogret et al., 2021), in particular, through the production of IL-21 (Niogret et al., 2021). While the proposed mechanisms in tumors and lymph nodes appear to be quite different, these studies collectively point out Tfh or Tfh-like cells as central players of PD-1 blockade, likely due to their high PD-1 expression and their specific cytokine/chemokine program.

Another unexpected observation was the critical role of IL-4 in mediating the effect of anti-PD-1 in the tumor-draining lymph node. Indeed, IL-4 was found to be both necessary and sufficient to promote CD8^+^ T cell responses following anti-PD-1 mAb treatment. Tfh cells are the major producers of IL-4 in secondary lymphoid organs (Glatman Zaretsky et al., 2009; King and Mohrs, 2009; Reinhardt et al., 2009; Vijayanand et al., 2012), and we confirmed that this was the case in tumor-draining lymph nodes. Consistently, inhibition of BCL6 prevented the upregulation of IL-4 in response to anti-PD-1 mAb. It is likely that the accumulation, activation, and redistribution of Tfh cells upon anti-PD-1 treatment favor CD8^+^ T cell sensing of IL-4 in the lymph node. Of note, a few studies have reported a role of IL-4 in promoting CD8^+^ T cell responses in other contexts (Carvalho et al., 2002; Morris et al., 2009; Oliver et al., 2012). We found that the addition of IL-4 is sufficient to enhance T cell expansion in vitro, suggesting that IL-4 acts directly on CD8^+^ T cells, although it remains possible that additional cell types respond to this cytokine. The role of IL-4 may be context dependent (including sites and therapies), as circulating IL-4 levels have been associated with better responses in anti-PD-1 mAb-treated non-small-cell lung carcinoma patients (Boutsikou et al., 2018), while other studies have suggested deleterious impacts of basal IL-4 in antitumor responses (Maier et al., 2020; Shirota et al., 2017).

In the context of vaccination with MVA, we found that anti-PD-1 increased the magnitude and quality of antigen-specific CD8^+^ T cell responses in the draining lymph node and even promoted the response of subdominant or cryptic epitopes. These effects were again dependent on IL-4. Our results may provide a mechanistic basis for the recent observation that anti-PD-1 mAb promotes flu, simian immunodeficiency virus), or tumor vaccine responses (Herati et al., 2022; Liu et al., 2022; Rahman et al., 2021) and increases circulating Tfh cells (Herati et al., 2022).

In sum, we describe here a general mechanism by which anti-PD-1 mAb promotes peripheral CD8^+^ T cell responses by targeting Tfh cells and inducing IL-4 production. Increasing Tfh cell numbers and associated cytokines represents an attractive goal to boost the peripheral activity of anti-PD-1 mAb in both tumor and vaccine settings.

## Materials and methods

### Mice

6–8-wk-old C57BL/6J mice were purchased from ENVIGO and housed at the animal care facility of the Pasteur Institute. Ubi-GFP-OT-I-Rag1^−/−^, OT-I-Rag1^−/−^, and CD11c-eYFP mice were bred in our animal facility. PD-1^−/−^ mice were purchased from the Jackson Laboratory and bred in our animal facility. All animal studies were approved by the Pasteur Institute Safety Committee in accordance with French and European Guidelines (Committee for Ethics in Animal Experimentation 170038 and 220108) and validated by the French Ministry of Education and Research (#43192-2023042711277493 v4 and #34664-2022011317403060 v1).

### BGRST humanized immune system mice

BGRST mice are *BALB/c Rag2*^*−/−*^ *IL2rg*^−/−^ *Sirpa*^*NOD*^ mice expressing the TLSP gene under the control of the K14 promoter (Li et al., 2018). Briefly, fetal liver CD34^+^ cells (Advanced Bioscience Resources Inc.) were isolated with affinity matrices according to the manufacturer’s instructions (Miltenyi Biotec) and subsequently phenotyped for CD34 and CD38 expression. Newborn (5–9 days old) pups received sublethal irradiation (2.8 Gy) and were injected intrahepatically with 9–16 × 10^4^ CD34^+^CD38^−^ human fetal liver cells. 12 wk after graft, reconstitution of the human immune system was confirmed by analysis of human CD45, CD19, and CD3 expression on live leukocytes in the blood using flow cytometry. Mice were injected intravenously with 250 µg of biosimilar nivolumab (BioXCell) or with PBS. Lymph nodes were collected 3 days after nivolumab injection, and cells were analyzed by flow cytometry.

### Tumor cell lines

The OVA-expressing EG7 tumor cell line was cultured in RPMI medium (Gibco) containing 10% heat-inactivated fetal bovine serum [FBS], penicillin (50 ml^−1^), streptomycin (50 µg.ml^−1^), 1 mM sodium pyruvate, 10 mM Hepes, 50 µM 2-mercaptoethanol, G418 (0.4 mg.ml^−1^), and hygromycin (0.2 mg.ml^−1^). MC38 cells (Kerafast) were transduced in our laboratory to express chicken OVA and cloned to generate an MC38-OVA cell line. The MC38-OVA cells were cultured in DMEM medium (Gibco) containing 10% heat-inactivated FBS, penicillin (50 ml^−1^), streptomycin (50 µg.ml^−1^), 1 mM sodium pyruvate, 10 mM Hepes, 1 mM non-essential amino acids, and 50 µM gentamicin. Cells were routinely monitored for mycoplasma contamination.

### Tumor implantation

Mice were subcutaneously injected in the right flank with EG7 or MC38-OVA (0.5 × 10^6^ tumor cells in 200 µl of PBS). Tumor volume was measured three times per week and mice were euthanized when humane endpoints were reached. The percentage of tumor growth was calculated using the following formula: (tumor volume − tumor volume at treatment initiation)/tumor volume at treatment initiation ×100.

### Treatments

Tumor-bearing mice were i.v. injected 10 days after tumor inoculation with 250 µg of anti-PD-1 mAb (clone RMP1-14, rat IgG2a; BioXCell), PBS, or 250 µg of a rat IgG2a control isotype (BioXCell). All experiments were controlled at least once with mice injected with a control isotype. Additional experiments using PBS-injected mice as a control group showed no detectable differences with isotype-treated mice in our read-out. To block lymphocyte egress from lymph nodes, mice were injected i.p. with 20 µg of FTY720 (Selleckchem). Mice were treated with FTY720 1 day prior to anti-PD-1 mAb treatment and then 5 days per week for 2 wk. To impair Tfh cell’s function, mice were injected i.p. with 25 µg of the BCL6 BTB domain inhibitor FX1 (Merck) (Cai et al., 2020, 2021; Cardenas et al., 2016) or with vehicle as a control. FX1 was dissolved in DMSO and then diluted in H_2_O cyclodextrin 1%. Mice were treated 1 day prior to anti-PD-1 mAb injection and then every 2 days until the end of the experiment. For survival experiments, FX1 treatment was stopped after 10 doses and survival continued to be monitored thereafter. To test the role of IL-4 signaling, tumor-bearing mice were injected i.p. with 250 µg anti-IL-4 (clone 11B11; BioXCell) or an IgG1,κ isotype 1 day prior anti-PD-1 mAb treatment followed by two injections of 150 µg at 18 and 36 h after anti-PD-1 injection. When indicated, mice were injected i.p. with IL-4 complexes prepared with 5 µg murine IL-4 (Peprotech) and 25 µg anti-IL-4 (clone 11B11; BioXCell) i.p. as described (Finkelman et al., 1993; Morris et al., 2009).

### Adoptive transfer

OT-I CD8^+^ T cells were isolated from Ubi-GFP-OT-1 TCR-Rag1^−/−^ transgenic mice, labeled with the Cell Trace Violet (CTV) Proliferation Dye (Thermo Fisher Scientific) according to the manufacturer’s instructions, and injected i.v. (3 × 10^6^ cells per mice).

### MVA vaccination

Recombinant MVA-HIV-B (expressing full-length HIV Gag, fused to three Pol and two Nef fragments) (Sagoo et al., 2016) was provided by the Agence Nationale de Recherche sur le Sida. Mice were injected into the footpad with 2 × 10^6^ p.f.u. of MVA-HIV-B.

### Fluorescently labeled anti-PD-1 antibody

Anti-PD-1 mAb was conjugated with Alexa Fluor 594 Conjugation kit (Fast)—Lightning-Link (Abcam) following the manufacturer’s instruction. Tumor-bearing mice were injected i.v with 250 μg labeled anti-PD-1 mAb (clone RMP1-14). In vivo binding of labeled anti-PD-1 Ab was assessed 20 h later by flow cytometry. A second anti-PD-1 clone (29F.1A12), whose staining was not blocked by prior RMP1-14 binding (Polesso et al., 2021), was used to stain lymph node cells ex vivo.

### In vitro assays

Naïve OT-I CD8^+^ T cells were labeled with the Cell Trace Violet Proliferation dye and activated using 0.5 nM SIINFEKL peptide with the addition of 20 ng/ml IL-15 at 24 h to increase cell survival. Cells were supplemented or not with 20 ng/ml IL-4 at 24 and 48 h. Proliferation was assessed on day 4.

### Flow cytometry

Single-cell suspensions were generated from tumor-draining lymph nodes, non-draining lymph nodes, and tumor tissue. For standard cell surface staining, single cells were stained in FACS buffer (2% FBS, 0.2% EDTA in PBS) in the presence of anti-CD16 antibody (1:200; BioLegend) and fixable viability dye eF780 (1:1,000; Invitrogen) for 1 h using the following antibodies: CD45.2-BUV737 (1:100, clone 104), NK1.1-BV650 (1:200, clone PK136), TCRβ-BV650 (1:200, clone H57-597), CD4-BUV395 (1:200, clone GK1.5), CD4-BUV805 (1:200, clone GK1.5), CD4-BV510 (1:200, clone RM4-5), and CD8α-BUV395 (1:200, clone 53-6.7), B220-BUV395 (1:100, clone RA3-6B2), and PDL-1-BUV395 (1:100, clone MIH5) purchased from BD Biosciences. Moreover, CD19-BV650 (1:200, clone 6D5), CD19-Alexa 647 (1:100, clone 6D5), NK1.1-BV711 (1:200, clone PK136), TCRβ-PE (1:200, clone H57-597), CD4-APC (1:200, clone GK1.5), CD4-Alexa488 (1:200, clone GK1.5), CD4-PE (1:200, clone GK1.5), CD8α-BV786 (1:200, clone 53-6.7), CD8-APC-fire750 (1:100, clone 53-7.1), CD44-BV605 (1:100, clone IM7), CD84-PE (1:100, clone mCD84.7), PD-1-PeCy7 (1:100, clone 29F.1A12), PD-1-BV421 (1:100, clone 29F.1A12), PD-1-PE-Dazzle594 (1:100, clone 29F.1A12), GL7-PerCPCy5.5 (1:100, clone GL7), IgD-APC (1:100, clone AMS), IL-4Rα-PeCy7 (1:100, clone I015F8) or IgG2b,κ-PeCy7 (1:100, clone TRK4530), CD25-PE (1:100, clone PC61), CD80-V605 (1:100, clone 16-10A1), and CD86-PE (1:100, clone GL-1) were purchased from BioLegend. Finally, CD69-PerCPCy5.5 (1:100, clone H1.2F3) was purchased from eBioscience.

For intracellular cytokine staining, cells were restimulated as indicated for 4 h in the presence of brefeldin A (1 μg/ml; BD Biosciences) and stained with IFNγ-BUV737 (1:100, clone XMG1.2) from BD Biosciences, and TNFα-Alexa647 (1:100, clone MP6-XT22), TNFα-Alexa488 (1:100, clone MP6-XT22), and IL-4-PE (1:50, clone 11B11) purchased from BioLegend. To examine the H2-K^b^-restricted MVA-specific CD8^+^ T cell responses, we used the following epitopes (Sagoo et al., 2016): A8R (189, ITYRFYLI), A3L (270–277, KSYNYMLL), B8R (20–27, TSYKFESV), K3L (6–15, YSLPNAGDVI), and A19L (47–55, VSLDYINTM) purchased from Polypeptide Laboratories. H2-K^b^-OVA_257-264_ and H2-K^b^-TSYKFESV MHC:peptide tetramers were kindly provided by the National Health Institute Tetramer Core Facility. Tetramer staining was performed first followed by staining with the Ab cocktail. For intracellular staining, cells were fixed and permeabilized with FoxP3/Transcription Factor Staining Buffer Set (Thermo Fisher Scientific). Antibodies for intracellular staining—BCL6-BUV615 (1:100, clone K112-91) or BCL6-Alexa647 (1:100, clone K112-91) from BD Biosciences; or Ki67-BV421 (1:100, clone 16A8) and IL-4-BV421 (1:100, 11B11) from BioLegend; and Ki67-eF660 (1:100, clone SolA15), FoxP3-PE (1:100, clone FJK-16 s), RORγτ− PE-eFluor610 (1:100, clone AFKJS-9), GATA3-PerCP-eFluor710 (1:100; TWA), and T-bet-APC (1:100; ebio-4B10) from eBioscience—were incubated overnight at 4°C. CXCR5 staining was performed using biotinylated-CXCR5 Ab (clone L138D7, 1:25; BioLegend) for 3 h at 4°C and revealed by Streptavidin-BUV395 (1:300; BioLegend) or Streptavidin-BV421 (1:300; BioLegend).

For p-STAT6 staining, lymph node cell suspensions were fixed in PFA 4% for 10 min at room temperature. Samples were then permeabilized and fixed by methanol 90% for 30 min at −20°C. Overnight staining was performed at 4°C with anti-p-STAT6 (Y641)-PE (1:50, stock at 50 µg/ml, clone D8S9Y) or isotype control Rabbit IgG-PE (1:100, stock at 100 µg/ml, clone DA1E) purchased from Cell Signaling.

Cells isolated from humanized mice were stained in FACS buffer (2% FBS, 0.2% EDTA in PBS) in the presence of True Stained FcX (1:200; BioLegend) and fixable viability dye UV (1:1,000; Invitrogen) for 1 h using the following antibodies: hCD20-BUV737 (1:50, clone 2H7), hCD3-BUV805 (1:50, clone UCHT1), hCD84-BV421 (1:50, clone 2G7), HLA-A, B, C-BV605 (1:50, clone W6/32), hCD4-BV786 (1:50, clone SK3), hCXCR5-FITC (1:25, clone RF8B2), and hCD8-APCCy7 (1:50, clone SK1) purchased from BD Biosciences. For intracellular staining, cells were fixed and permeabilized with a FoxP3/Transcription Factor Staining Buffer Set (Thermo Fisher Scientific). For intracellular staining, BCL6-Alexa647 (1:50, clone K112-91) and Ki67-PE (1:50, clone B56) were purchased from BD Biosciences and incubated overnight at 4°C.

Counting beads (Invitrogen) were added to the samples to quantify absolute cell numbers. Data were acquired using a Fortessa or a Symphony flow cytometer (BD Biosciences) and analyzed using FlowJo software v10.8.1 (BD Biosciences). Cell sorting was performed using a BD FACS-Aria III Flow Cytometer (BD Bioscience).

### Multiplex assays for cytokine quantification

Cells (1 × 10^7^ cells) were resuspended in 400 µl of Lysis Buffer (Invitrogen) supplemented with (1:100) Protease Cocktail Inhibitor (Thermo Fisher Scientific). Pierce Universal Nuclease for Cell Lysis (Thermo Fisher Scientific) was added to each sample (1 µl per sample). Samples were incubated for 5 min on ice and sonicated briefly (30 s at 25 Hz). Supernatant was extracted by ultracentrifugation in a cold microfuge (14,000 *g* for 10 min). Multiplex assays were performed using 21-Plex ProcartPlex Panel (Invitrogen) following the manufacturer’s instructions. Analyses were performed using a Bio-Plex 200 system equipped with Bio-Plex Manager software (Bio-Rad).

### Two-photon imaging

Two-photon imaging of intact lymph nodes was performed as described (Beuneu et al., 2010) using CD11c-eYFP tumor-bearing hosts adoptively transferred using GFP-expressing OT-I CD8^+^ T cells. Two-photon imaging was performed with an upright microscope FVMPE-RS (Olympus) and a 25×/1.05 numerical aperture water-dipping objective (Olympus). Excitation was provided by an InSight DeepSee dual laser (Spectra Physics) tuned at 820 nm. To create time-lapse sequences, we typically scanned a 63-μm-thick volume with 7-μm Z-steps every 30 s. The following filters were used for fluorescence detection: YFP (542/27) and GFP (512/25). Movies were processed and analyzed with Fiji and Imaris software. Movies were anonymized and analysis was blinded and performed by two different laboratory members. Movies and figures are shown as two-dimensional maximum-intensity projections of 3D data.

### Lymph node immunofluorescence

Multiplex immunofluorescence imaging was performed using a PhenoCycler (Akoya Bioscences). Tumor-draining lymph nodes were fixed in 4% formaldehyde overnight, washed twice in PBS, and dehydrated using sucrose 30% at 4°C. Lymph nodes were embedded in OCT (Sakura Finetek). Samples were then flash-frozen using nitrogen-cooled isopentane. 10-micron tissue sections were mounted on poly-L-lysine (Sigma-Aldrich)–coated 22 × 22-mm glass coverslips (Electron Microscopy Sciences). Samples on coverslips were then prepared for PhenoCycler imaging according to the manufacturer’s instructions (Akoya Biosciences). Briefly, samples were fixed using 1% paraformaldehyde, washed, and stained for 3 h using a mixture of nucleotide-barcoded antibodies among: anti-mouse CD4-BX026 (clone RM4-5; Akoya Biosciences), anti-mouse CD19-BX020 (clone 6D5; Akoya Biosciences), anti-mouse Ki67-BX047 (clone B56; Akoya Biosciences), anti-mouse CD8a-BX029 (clone 53-6.7; Akoya Biosciences), anti-mouse FoxP3-BX004 (purified clone FJK-16 s from Thermo Fisher Scientific, barcoded in-house), anti-mouse CD86-BX006 (purified clone GL-1 from BioLegend, barcoded in house), and anti-mouse PD1-BX036 (purified clone 29F.1A12 from BioLegend, barcoded in house). Revealing of anti-CD4-BX026, anti-CD19-BX020, anti-Ki67-BX047, anti-CD8a-BX029, anti-FoxP3-BX004, anti-CD86-BX006, and anti-PD1-BX036 barcoded antibodies was performed using fluorophore-tagged complementary nucleotide sequences RX026-Atto550, RX20-Atto550, RX047-Atto550, RX029-Atto550, RX004-AlexaFluor488, RX006-Cy5, and RX036-Cy5, respectively. Image acquisition was carried out using the fluorescence Keyence BZ-X810 microscope equipped with a 20× air objective. Following PhenoCycler acquisition, images were processed using the PhenoCycler processor software (Akoya Biosciences). Cell segmentation was performed on QuPath (Bankhead et al., 2017) and using the StarDist cell segmentation script (Schmidt et al., 2018, *Preprint*). CSV files containing single-cell measurements of mean fluorescent intensities for each marker were then loaded on the spatial analysis software CytoMAP (Stoltzfus et al., 2020) on which cell phenotyping and distance analysis were carried out.

### Quantification of cell proliferation

Each generation of cells was identified using the *Proliferation tool* of Flow Jo v. 10.8.1. The replication index was the mean number of daughter cells for cells that divided at least once. The replication index was calculated using the following formula: $Replication\;index=\frac{\sum_{1}^{i}N_{i}}{\sum_{1}^{i}\frac{N_{i}}{2^{i}}}$ where *i* is the generation of cells and *N*_*i*_ is the number of cells in each generation (Roederer, 2011).

### Statistical analysis

All statistical tests were performed with Prism v.9.5.0 (GraphPad). Data are expressed as mean ± SEM. We used *t* test for two-group comparison and two-way analysis of variance (ANOVA) for multiple comparison. For mice survival, statistical analysis was performed using a log-rank test. All statistical tests were two-tailed with a significance level of 0.05. *P < 0.05, **P < 0.01, ***P < 0.001; ns, non-significant.

### Online supplemental material

Fig. S1 shows the impact of anti-PD-1 therapy in tumor-draining lymph node in the EG7 tumor model. Fig. S2 evaluates the consequences of anti-PD-1 mAb on lymph node DCs. Fig. S3 shows the binding of fluorescent anti-PD-1 mAb on lymph node cells. Fig. S4 reports the impact of anti-PD-1 mAb on various immune cells in the lymph node. Fig. S5 shows the role of Tfh-derived IL-4 in the activity of anti-PD-1 mAb in lymph nodes. Video 1 shows the formation of CD8^+^ T cell–DCs interactions in tumor-draining lymph nodes.

## Data Availability

The data are available from the corresponding author upon reasonable request.
